# CCDC170 affects breast cancer apoptosis through IRE1 pathway

**DOI:** 10.18632/aging.202315

**Published:** 2020-12-03

**Authors:** Qiong Wang, Yanrui Zhao, Hong Zheng, Qinghua Wang, Wei Wang, Ben Liu, Hongwei Han, Lina Zhang, Kexin Chen

**Affiliations:** 1 Department of Epidemiology and Biostatistics, Tianjin Medical University Cancer Institute and Hospital, National Clinical Research Center for Cancer, Tianjin 300060, P.R. China; 2 Key Laboratory of Cancer Prevention and Therapy, Tianjin 300060, P.R. China; 3 Tianjin’s Clinical Research Center for Cancer, Tianjin 300060, P.R. China; 4 Key Laboratory of Molecular Cancer Epidemiology, Tianjin 300060, P.R. China; 5 Key Laboratory of Breast Cancer Prevention and Therapy, Tianjin Medical University, Ministry of Education, Tianjin 300060, P.R. China; 6 Department of Breast Oncology, Tianjin Medical University Cancer Institute and Hospital, Tianjin 300060, P.R. China

**Keywords:** breast cancer, CCDC170, IRE1α, XBP1, apoptosis

## Abstract

Genome-wide association studies have revealed that multiple single-nucleotide polymorphisms in the intergenic region between estrogen receptor 1 and coiled-coil domain containing 170 (*CCDC170*) are associated with breast cancer risk. We performed microarray and bioinformatics analyses to identify genes that were induced upon CCDC170 overexpression, and confirmed our findings by evaluating paraffin-embedded breast cancer tissues and conducting cellular assays. In CCDC170-overexpressing MCF7 breast cancer cells, microarray analyses revealed that inositol-requiring enzyme 1 (*IRE1*) was the most elevated gene in enriched pathways. In breast cancer tissues, IRE1 expression correlated positively with CCDC170 and X-box binding protein 1 expression at both the mRNA and protein levels. In a survival analysis, patients with higher CCDC170 levels exhibited better disease-free survival. Western blotting indicated that overexpressing CCDC170 in MCF7 cells increased protein levels of IRE1α, estrogen receptor α and X-box binding protein 1, while silencing CCDC170 reduced them. CCDC170 overexpression promoted apoptosis in MCF7 cells, and this effect was more obvious under endoplasmic reticulum stress. MCF7 cells overexpressing CCDC170 were more sensitive to paclitaxel. Our study showed that higher CCDC170 expression is associated with a better prognosis in breast cancer patients and that CCDC170 may promote apoptosis through the IRE1α pathway.

## INTRODUCTION

Breast cancer is one of the most common malignant tumors threatening women’s health worldwide [1]. Environmental and genetic factors are known to influence breast cancer risk. Hereditary breast cancer accounts for 5-10% of all breast cancer cases, and pathogenic variants in the *BRCA1/2* genes have been detected in approximately 90% of hereditary breast cancer cases [2, 3]. In addition, many genetic polymorphisms have been reported to be associated with breast cancer risk [4]. Genome-wide association studies have revealed that single-nucleotide polymorphisms residing in the intergenic region between estrogen receptor 1 (*ESR1*) and coiled-coil domain containing 170 (*CCDC170*) at 6q25.1 are associated with breast cancer risk. While the function of *ESR1* in breast cancer has been well studied, that of *CCDC170* remains elusive [5].

*CCDC170*, also known as *C6orf97*, is a largely uncharacterized open reading frame located about 69 kilobases upstream of *ESR1* on 6q25.1, and spanning around 127 kilobases (http://genome.ucsc.edu/, [GRCh38/hg38]). *CCDC170* was found to be co-expressed with *ESR1* in breast cancer tissues [6], and an *ESR1*-*CCDC170* rearrangement was discovered in luminal B breast tumors [7]. ERα, the protein encoded by *ESR1*, is well known to be an independent prognostic factor in breast cancer, and thus is a target of endocrine therapy [8]. We speculated that *CCDC170* might have important functions and clinical significance in breast cancer, considering its relationship with *ESR1*.

Inositol-requiring enzyme 1 alpha (IRE1α), encoded by *IRE1*, is an essential signal transducer in the most conserved unfolded protein response signaling branch. IRE1α has multiple functions, including unconventionally shearing X-box binding protein 1 (*XBP1*), triggering apoptosis and inducing autophagy [9–12]. Upon the activation of the unfolded protein response, IRE1α cleaves 26 nucleotides from the unspliced *XBP1* mRNA sequence, yielding a spliced form that encodes the active transcription factor XBP1s. XBP1s subsequently enhances the expression of genes involved in protein folding and degradation [10]. Notably, *ESR1* and *XBP1* are co-expressed in breast cancer tissues [13, 14], and XBP1s can bind to the X-box in the *ESR1* promoter, thus inducing its transcription [15, 16].

Apoptosis is one of the principal mechanisms used to induce cell death in cancer therapeutics. Tumor resistance occurs when tumor cells adapt to therapy by activating anti-apoptotic pathways and altering the tumor microenvironment [17]. Apoptotic cell death following the unfolded protein response depends primarily on IRE1α [18]. Thus, the IRE1α-XBP1 pathway has been an attractive cancer therapeutic target [19, 20], and has been identified as a vital determinant of drug resistance [21–24].

In this study, we constructed a gene co-expression network to identify genes that might be associated with CCDC170 function. Our findings suggested the existence of a regulatory loop involving CCDC170, IRE1α, XBP1 and ERα. Thus, we investigated the effects of CCDC170 on apoptosis, and examined whether CCDC170 activity depended on the IRE1α pathway.

## RESULTS

### The influence of CCDC170 on the gene expression profile of MCF7 cells

**Figure 1:**
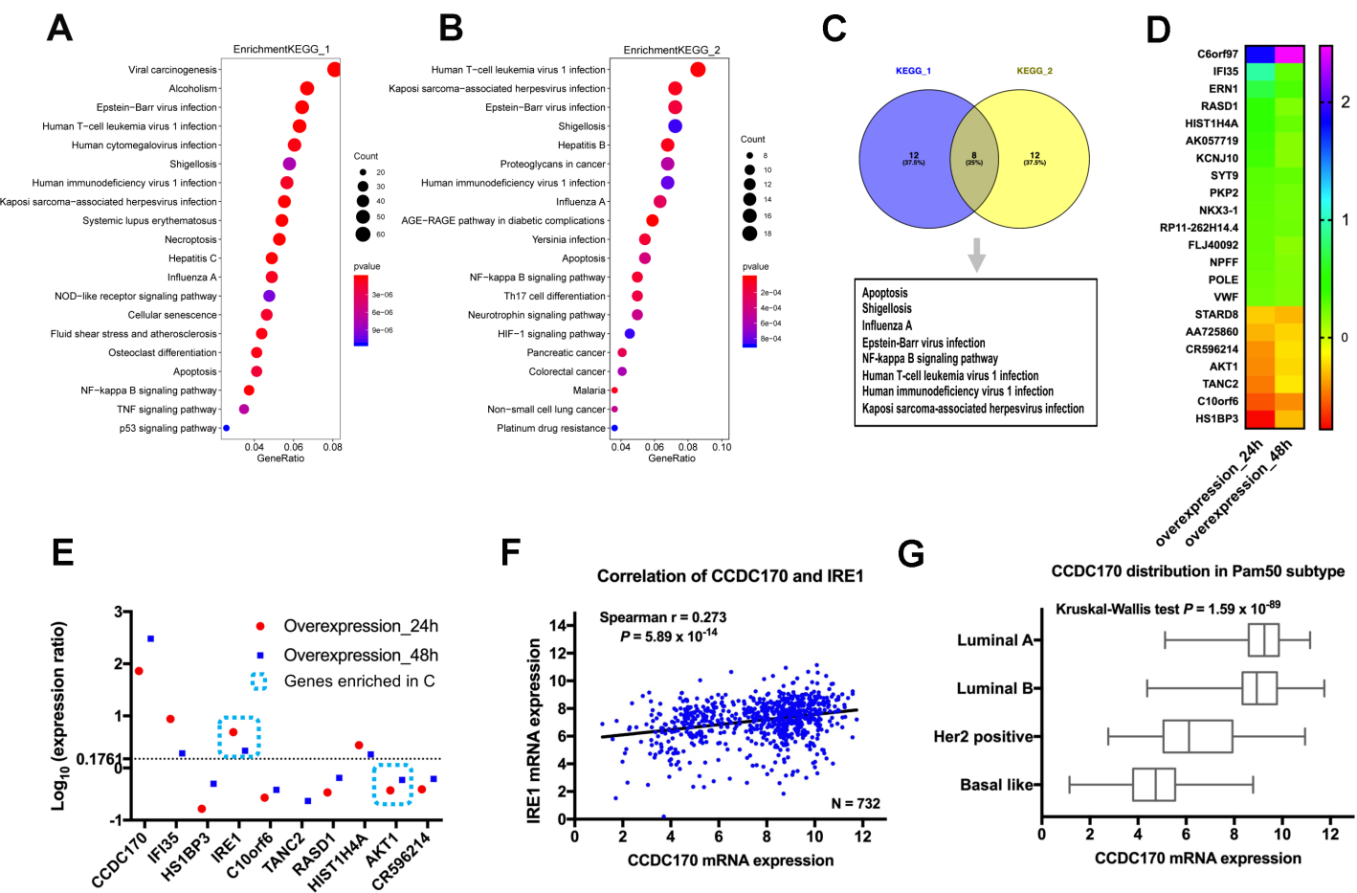
**The influence of CCDC170 on the gene expression profile of MCF7 cells.** The top 20 significantly enriched pathways in 24-hour group (**A**) and 48-hour group (**B**) of CCDC170 upregulation in MCF7 breast cancer cells. Gene Ratio represented the ratio of the DEGs number to the total gene number in a certain pathway. The color of the *P* values indicated the significance of the Gene Ratio. The size of the circle indicated the number of the target genes involved in a certain pathway. (**C**) The eight overlapping enriched pathways both in 24-hour group and 48-hour group of CCDC170 overexpression. (**D**) The top 20 DEGs with consistent expression-change tendencies in the 24-hour and 48-hour groups. (**E**) The expression ratio of the top 10 DEGs with consistent expression-change tendencies in the 24-hour and 48-hour groups. Only *IRE1* and *AKT1* were involved in the eight overlapping pathways, and the change of *IRE1* expression was the most obvious. (**F**) Positive correlation between *CCDC170* and *IRE1* levels (r = 0.273, *P* = 5.89×10^-14^). (**G**) The differential distribution of *CCDC170* expression in Pam50 subtypes (*P* = 1.59×10^-89^). KEGG, Kyoto Encyclopedia of Genes and Genomes. DEGs, differentially expressed genes. Datasets from TCGA were implemented for the correlation and distribution analysis.

To identify genes that could be induced by CCDC170, we transfected MCF7 breast cancer cells with either a pCMV-N-Flag-CCDC170 vector or a pCMV-N-Flag control vector. We extracted mRNA from the cells 24 or 48 hours post-transfection, and used microarrays to assess their gene expression profiles. Then, we performed an enrichment analysis of the differentially expressed genes (DEGs) between the CCDC170-overexpressing group and the control group. When we compared the top 20 significantly enriched pathways between the 24-hour group (Figure 1A) and the 48-hour group (Figure 1B), we identified eight common pathways (Figure 1C). We also determined the top 20 genes with consistent expression-change tendencies in the 24- and 48-hour groups (Figure 1D). Among these genes, only *IRE1* and *AKT1* were involved in the eight pathways mentioned above. The change in *IRE1* expression (24h: log[fold ratio] = 0.69; 48h: log[fold ratio] = 0.33) was the most obvious in the apoptotic pathway (Figure 1E).

Next, we determined the correlation between *CCDC170* and *IRE1* expression in breast cancer tissues from The Cancer Genome Atlas (TCGA) and the Gene Expression Omnibus (GEO). There was a significant positive correlation between *CCDC170* and *IRE1* levels in TCGA (r = 0.273, *P* = 5.89×10^-14^; (Figure 1F), as well as in GEO (r = 0.262, *P* = 9.19×10^-55^; (Supplementary Figure 1A). When cases in TCGA were stratified by subtype, *CCDC170* mRNA levels were higher in the luminal A and luminal B subtypes, but were lower in the human epidermal growth factor receptor 2 (Her2)-positive and Basal-like breast cancer subtypes (Figure 1G). A similar tendency was found in the GEO database (Supplementary Figure 2F).

### The expression of CCDC170, IRE1α and XBP1s in breast cancer tissues

**Figure 2:**
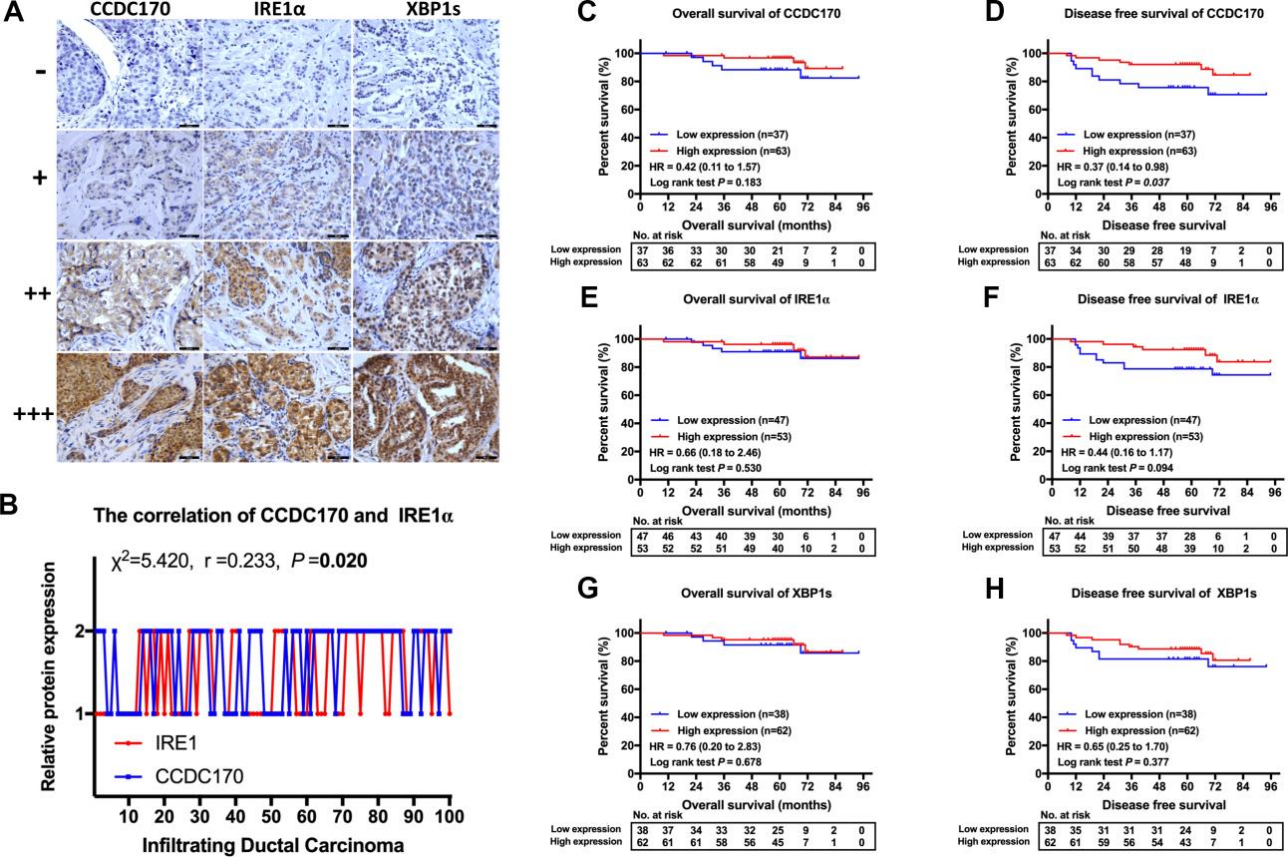
**IHC stain and the prognosis value of CCDC170, IRE1α and XBP1s in breast cancer tissues.** (**A**) Representative IHC staining of CCDC170, IRE1α and XBP1s. Scale bar: 50μm. (**B**) The correlation between CCDC170 and IRE1α levels in breast cancer tissues (r = 0.233, *P* = 0.020). 1, 2 represented low expression (0-2 staining index) and high expression (3-12 staining index) respectively. n = 100 cases. (**C, D**) The CCDC170 high-expression group exhibited better DFS (*P* = 0.037), but no significance in OS (*P* = 0.183). (**E, F**) The expression of IRE1α showed no significance in OS (*P* = 0.530) and DFS (*P* = 0.094). (**G, H**) The expression of XBP1s showed no significance in OS (*P* = 0.678) and DFS (*P* = 0.377).

To investigate the prognostic value of CCDC170, IRE1α and XBP1s in breast cancer, we used immunohistochemistry to assess the expression of these proteins in 100 patients with invasive ductal carcinoma. General information about the included breast cancer patients can be found in Supplementary Table 1. IRE1α levels were found to correlate with CCDC170 levels (r = 0.233, *P* = 0.020; Figure 2B) and XBP1s levels (r = 0.212, *P* = 0.034; Table 1). Additionally, CCDC170 levels correlated positively with XBP1s levels (r = 0.339, *P* = 0.001; Table 1). In terms of clinicopathological characteristics, CCDC170 levels correlated positively with ERα levels (r = 0.389, *P* = 9.90×10^-5^). IRE1α levels correlated positively with Her2 levels (r = 0.293, *P* = 0.003) and Ki67 levels (r = 0.208, *P* = 0.038). XBP1s levels exhibited a notable positive correlation with ERα levels (r = 0.286, *P* = 0.004). Moreover, CCDC170 levels (r = 0.333, *P* = 0.006) and IRE1α levels (r = 0.353, *P* = 0.003) correlated with the molecular subtypes. However, CCDC170, IRE1α and XBP1s levels were not significantly associated with the tumor size, lymph node status, Tumor-Node-Metastasis (TNM) stage or histologic grade (Table 1). Based on these findings, we explored the correlation of *CCDC170* levels with *XBP1* and *ESR1* levels in TCGA and the GEO database. In TCGA, *CCDC170* levels were positively associated with *XBP1* levels (r = 0.636, *P* = 2.42×10^-84^; (Supplementary Figure 3A) and *ESR1* levels (r = 0.805, *P* = 1.34×10^-167^; (Supplementary Figure 3B). Similarly, *CCDC170* levels were positively associated with *XBP1* levels (r = 0.601, *P* = 0.000; (Supplementary Figure 1B) and *ESR1* levels (r = 0.747, *P* = 0.000; (Supplementary Figure 1C) in the GEO database. In addition, a positive correlation between IRE1 expression and XBP1 expression was found in both TCGA (r = 0.291, *P* = 1.06×10^-15^; (Supplementary Figure 3C) and GEO (r = 0.244, *P* = 1.48×10^-47^; (Supplementary Figure 1D) database. We also analyzed the associations of *CCDC170*, *IRE1* and *XBP1* mRNA levels with clinicopathologic characteristics such as ERα, progesterone receptor (PR), Her2 and Ki67 levels, the PAM50 subtype, tumor size, lymph node status and TNM stage in TCGA (Supplementary Figures 3D, 3E, 4A–4R) and GEO (Supplementary Figure 2A–2R). The details of these correlations are presented in Supplementary Figures 2, 4.

**Table 1:** The relationship between CCDC170, IRE1α, XBP1s and clinical pathological features.

**Feature**		**CCDC170, N (%)**		**χ^2^**	**r**	***P***	**IRE1α, N (%)**		**χ^2^**	**r**	***P***	**XBP1s, N (%)**		**χ^2^**	**r**	***P***
		**Low**	**High**				**Low**	**High**				**Low**	**High**			
Tumor size																
(d/cm)	≤ 2	12 (31.6)	26 (68.4)	0.773	-0.088	0.379	16 (43.1)	22 (57.9)	0.589	-0.077	0.443	11 (28.9)	27 (71.7)	2.132	-0.146	0.144
	>2	25 (40.3)	37 (59.7)				31 (50.0)	31 (50.0)				27 (43.5)	35 (56.5)			
Lymph node																
metastasis	No	17 (34.7)	32 (65.3)	0.219	-0.047	0.640	19 (38.8)	30 (61.2)	2.609	-0.162	0.106	21 (42.9)	28 (57.1)	0.962	0.098	0.327
	Yes	20 (39.2)	31 (60.8)				28 (54.9)	23 (45.1)				17 (33.3)	34 (66.7)			
TNM stage																
	I	11 (32.4)	23 (67.6)	0.525	-0.048	0.769	13 (38.2)	21 (61.8)	1.866	-0.135	0.393	11 (32.4)	23 (67.6)	2.838	0.003	0.242
	II	15 (40.5)	22 (59.5)				18 (48.6)	19 (51.4)				18 (48.6)	19 (51.4)			
	III	11 (37.9)	18 (62.1)				16 (55.2)	13 (44.8)				9 (31.0)	20 (69.0)			
Grade																
	I	0 (0.0)	5 (100.0)	5.131	-0.230	0.077	0 (0.0)	5 (100.0)	4.611	-0.142	0.100	1 (20.0)	4 (80.0)	3.042	-0.190	0.218
	II	24 (39.3)	37 (60.7)				30 (49.2)	31 (50.8)				22 (36.1)	39 (63.9)			
	III	10 (55.6)	8 (44.4)				9 (50.0)	9 (50.0)				10 (55.6)	8 (44.4)			
ER																
	-	26 (57.8)	19 (42.2)	15.15	0.389	**9.90x10^-5^**	23 (51.1)	22 (48.9)	0.555	0.075	0.456	24 (53.3)	21 (46.7)	8.165	0.286	**0.004**
	+	11 (20.0)	44 (80.0)				24 (43.6)	31 (56.4)				14 (25.5)	41 (74.5)			
PR																
	-	25 (43.9)	32 (56.1)	2.676	0.164	0.102	26 (45.6)	31 (54.4)	0.102	-0.032	0.749	23 (40.4)	34 (59.6)	0.311	0.056	0.577
	+	12 (27.9)	31 (72.1)				21 (48.8)	22 (51.2)				15 (34.9)	28 (65.1)			
Her-2																
	-	27 (38.0)	44 (62.0)	0.111	0.033	0.739	40 (56.3)	31 (43.7)	8.570	0.293	**0.003**	26 (36.6)	45 (63.4)	0.198	-0.044	0.656
	+	10 (34.5)	19 (65.5)				7 (24.1)	22 (75.9)				12 (41.4)	17 (58.6)			
Ki-67																
	≤ 14%	6 (31.6)	13 (68.4)	0.296	-0.054	0.587	13 (68.4)	6 (31.6)	4.321	0.208	**0.038**	6 (31.6)	13 (68.4)	0.410	-0.064	0.522
	>14%	31 (38.3)	50 (61.7)				34 (42.0)	47 (58.0)				32 (39.5)	49 (60.5)			
IRE1α																
	-	23 (48.9)	24 (51.1)	5.420	0.233	**0.020**						23 (48.9)	24 (51.1)	4.502	0.212	**0.034**
	+	14 (26.4)	39 (73.6)									15 (28.3)	38 (71.7)			
XBP1s																
	-	22 (57.9)	16 (42.1)	11.480	0.339	**0.001**	23 (60.5)	15 (39.5)	4.502	0.212	**0.034**					
	+	15 (24.2)	47 (75.8)				24 (38.7)	38 (61.3)								
CCDC170																
	-						23 (62.2)	14 (37.8)	5.420	0.233	**0.020**	22 (59.5)	15 (40.5)	11.480	0.339	**0.001**
	+						24 (38.1)	39 (61.9)				16 (25.4)	47 (74.6)			
Molecular																
subtype	Luminal A	4 (25.0)	12 (75.0)	12.484	0.333	**0.006**	11 (68.8)	5 (31.3)	14.198	0.353	**0.003**	4 (25.0)	12 (75.0)	7.501	0.264	0.058
	Luminal B	12 (25.0)	36 (75.0)				18 (37.5)	30 (62.5)				14 (29.2)	34 (70.8)			
	HER2	7 (46.7)	8 (53.3)				3 (20.0)	12 (80.0)				8 (53.3)	7 (46.7)			
	TNBC	14 (66.7)	7 (33.3)				15 (71.4)	6 (28.6)				12 (57.1)	9 (42.9)			

ER, estrogen receptor α; PR, progesterone receptor; Her-2, human epidermal growth factor receptor 2; TNBC, triple negative breast cancer; HER2, Her-2 overexpressed breast cancer; -, negative; +, positive.

### Survival analysis based on CCDC170, IRE1α and XBP1s expression

Next, we performed Kaplan-Meier analyses in our 100 patients according to their expression of CCDC170, IRE1α and XBP1s. The CCDC170 high-expression group exhibited better disease-free survival (DFS) than the low-expression group (hazard ratio [HR] [95% confidence interval (CI)] = 0.37 [0.14 - 0.98], *P* = 0.037; Figure 2D). However, there was no significant difference in overall survival (OS) between the CCDC170 low- and high-expression groups (HR [95% CI] = 0.42 [0.11 - 1.57], *P* = 0.183; Figure 2C). We did not find differences in DFS or OS between the high and low-expression groups for IRE1α (Figure 2E, 2F) or XBP1s (Figure 2G, 2H). Considering that our patients had received different post-operative treatments, we also analyzed the association between CCDC170 expression and prognosis within cohorts that had received the same treatment. The results indicated that higher CCDC170 expression predicted better DFS in the chemotherapy cohort (Supplementary Figure 5D) and the radiation cohort (Supplementary Figure 5H), and predicted better OS in the radiation cohort (Supplementary Figure 5G).

We also performed Kaplan-Meier analyses using mRNA expression data from TCGA and GEO. In the GEO database, the high-expression groups for *CCDC170* (HR [95% CI] = 0.59 [0.47 - 0.73], *P* = 8.81×10^-7^; (Supplementary Figure 1E), *IRE1* (HR [95% CI] = 0.74 [0.60 - 0.91], *P* = 4.52×10^-3^; Supplementary Figure 1F) and *XBP1* (HR [95% CI] = 0.59 [0.48 - 0.73], *P* = 1.00×10^-6^;(Supplementary Figure 1G) exhibited better OS than their respective low-expression groups (based on the median). However, in TCGA, the expression of these genes was not significantly associated with the OS or DFS (Supplementary Figure 4S–4X).

### CCDC170 activated IRE1α/XBP1s signaling

**Figure 3:**
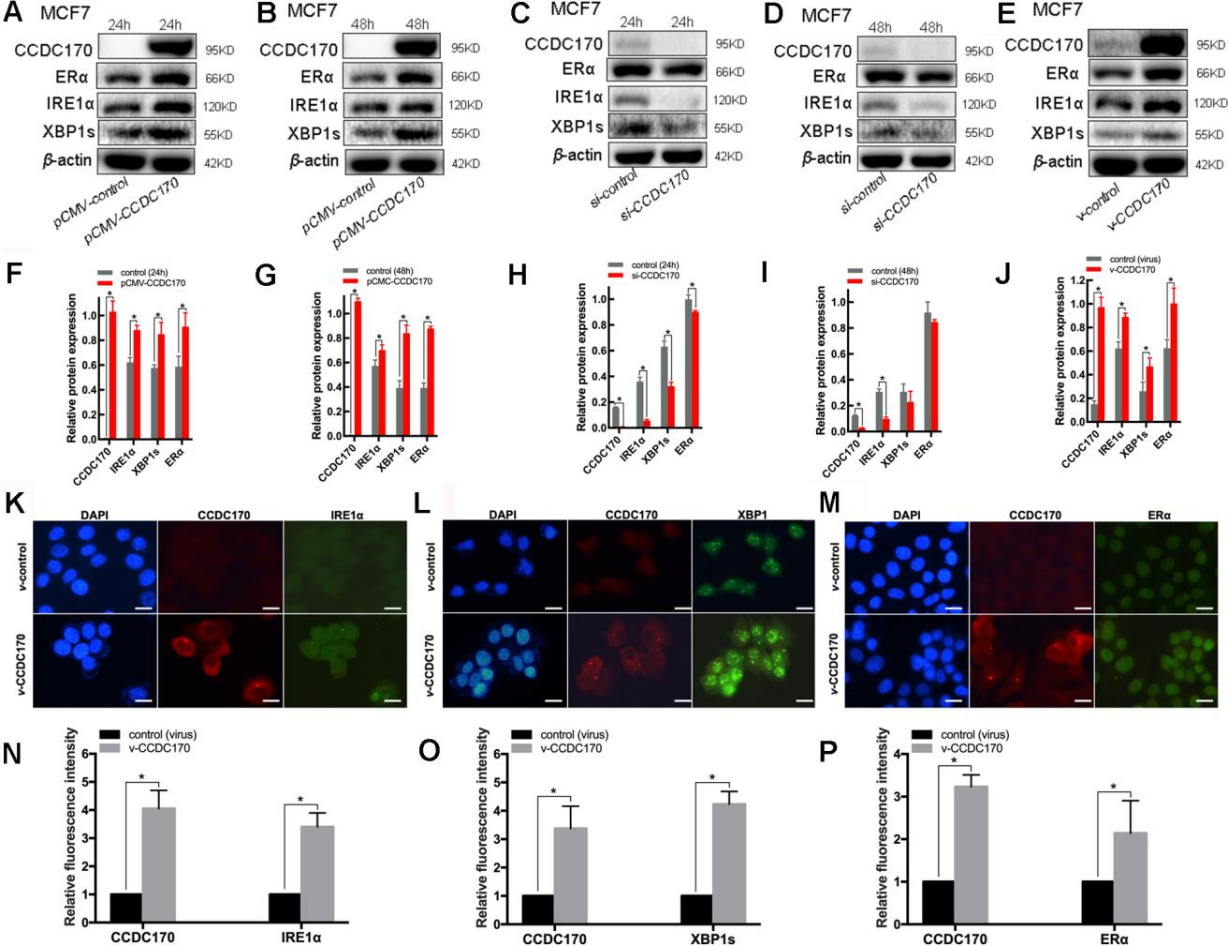
**The protein expression of CCDC170, IRE1α and XBP1s in MCF7 breast cancer cells.** Representative western blot bands and analysis at 24h (**A, F**) and 48h (**B, G**) of CCDC170 up-regulation, 24h (**C, H**) and 48h (**D, I**) of CCDC170 down-regulation. Representative western blot bands (**E**) and analysis (**J**) in MCF7 breast cancer cells that stably overexpressed CCDC170. *β*-actin was used as a reference for calculating the relative protein expression. Representative immunofluorescence images and analysis of IRE1α (**K, N**), XBP1s (**L, O**) and ERα (**M**, **P**) in CCDC170-stably-overexpressing MCF7 cells. Scale bar: 50μm. pCMV-CCDC170(control) represented CCDC170-transiently-overexpressing MCF7 cells and controls. v-CCDC170(control) represented CCDC170-stably-overexpressing MCF7 cells and controls. si-CCDC170(control) represented cells with siRNA-mediated knockdown of CCDC170 and the controls. The error bars presented as mean ± Standard Error of Mean (SEM) with analysis of unpaired Student’s t-test. *P < 0.05, compared with control group.

To further explore the relationship of CCDC170 with IRE1α, XBP1s and ERα, we conducted a series of *in vitro* assays in MCF7 breast cancer cells in which CCDC170 was overexpressed or silenced. Western blot analyses indicated that CCDC170 overexpression for 24 hours (Figure 3A, 3F) or 48 hours (Figure 3B, 3G) induced the expression of IRE1α (24h: *P =* 0.001; 48h: *P =* 0.025). CCDC170 overexpression also obviously increased the expression of ERα (24h: *P =* 0.009; 48h: *P =* 9.08×10^-4^) and XBP1s (24h: *P =* 0.016; 48h: *P =* 3.96×10^-5^). On the other hand, when CCDC170 was knocked down for 24 hours (Figure 3C, 3H) or 48 hours (Figure 3D, 3I), the expression of IRE1α decreased significantly (24h: *P =* 1.08×10^-4^; 48h: *P =* 1.69×10^-4^). However, the expression of XBP1s (24h: *P =* 5.05×10^-4^; 48h: *P =* 0.255) and ERα (24h: *P =* 0.010; 48h: *P =* 0.202) only decreased significantly at the 24-hour time point.

We also examined the effects of stably overexpressing CCDC170 in MCF7 breast cancer cells. When CCDC170 was continuously upregulated, the levels of IRE1α (*P =* 0.002), XBP1s (*P =* 0.026) and ERα (*P =* 0.012) all increased significantly (Figure 3E, 3J). Consistent with our microarray and Western blotting results, immunofluorescence analyses demonstrated that the fluorescence signals of IRE1α (*P =* 0.001; Figure 3K, 3N), XBP1s (*P =* 2.42×10^-4^; Figure 3L, 3O) and ERα (*P =* 0.041; Figure 3M, 3P) became stronger in MCF7 cells stably overexpressing CCDC170.

### CCDC170 promoted apoptosis and enhanced the sensitivity of MCF7 cells to paclitaxel

**Figure 4:**
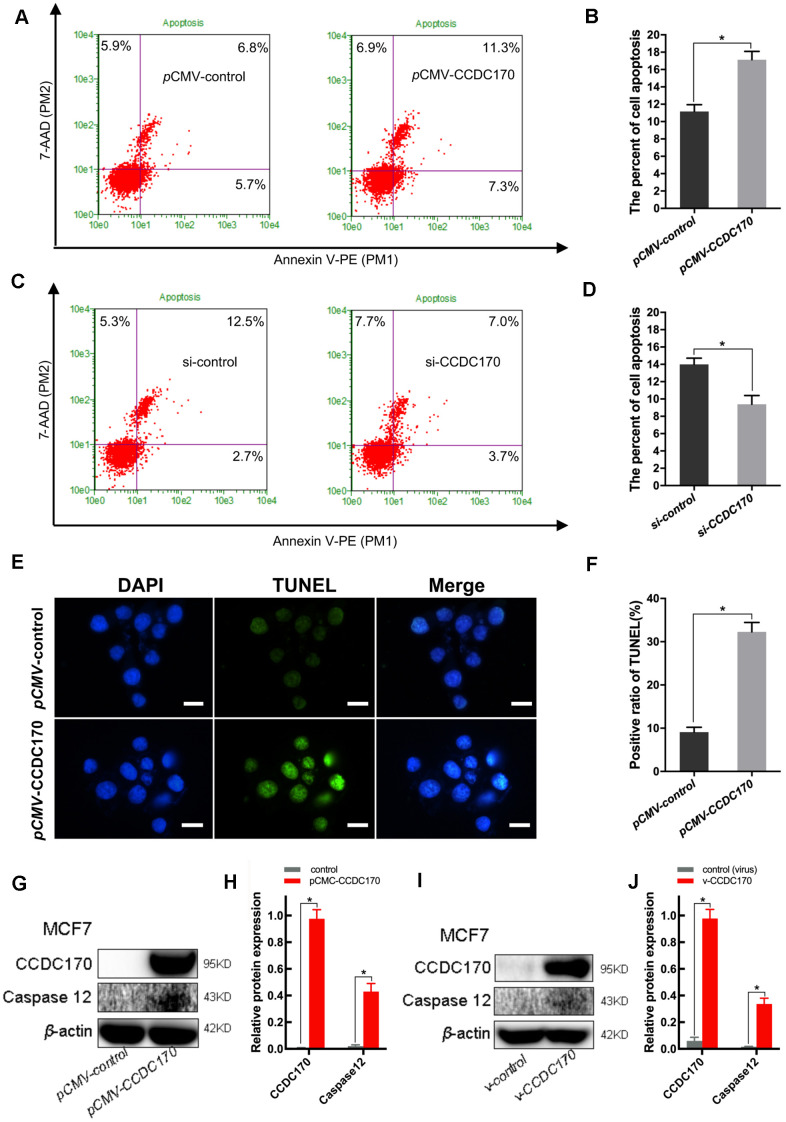
**CCDC170 promoted cell apoptosis in MCF7 breast cancer cells.** (**A, C**) Representative images of flow cytometry (FCM) using Annexin V-FITC and PI staining. Column bar graph showing a dramatically bigger early and late apoptosis ratio in CCDC170-transiently-overexpressing MCF7 cells than the control cells (**B**). The cell apoptosis ratio was significantly lower in the cells with CCDC170 knockdown compared with the control cells (**D**). Each group was independently repeated three times, 3000 cells were calculated. (**E**) Representative images were taken with nuclear stain DAPI (blue) and apoptosis stain TUNEL (green). (**F**) The result depicts the percentage of TUNEL positive nuclei of MCF-7 cells after CCDC170 upregulation. Scale bar, 50 μm. (**G, I**) Representative western blot bands of Caspase12 in MCF7 cells with CCDC170 up-regulated transiently (**H**) and stably (**J**). pCMV-CCDC170(control) represented CCDC170-transiently-overexpressing MCF7 cells and controls. v-CCDC170(control) represented CCDC170-stably-overexpressing MCF7 cells and controls. β-actin was used as a reference for calculating the relative protein expression. The error bars presented as mean ± Standard Error of Mean (SEM) with analysis of unpaired Student’s t-test. *P < 0.05, compared with control group.

**Figure 5:**
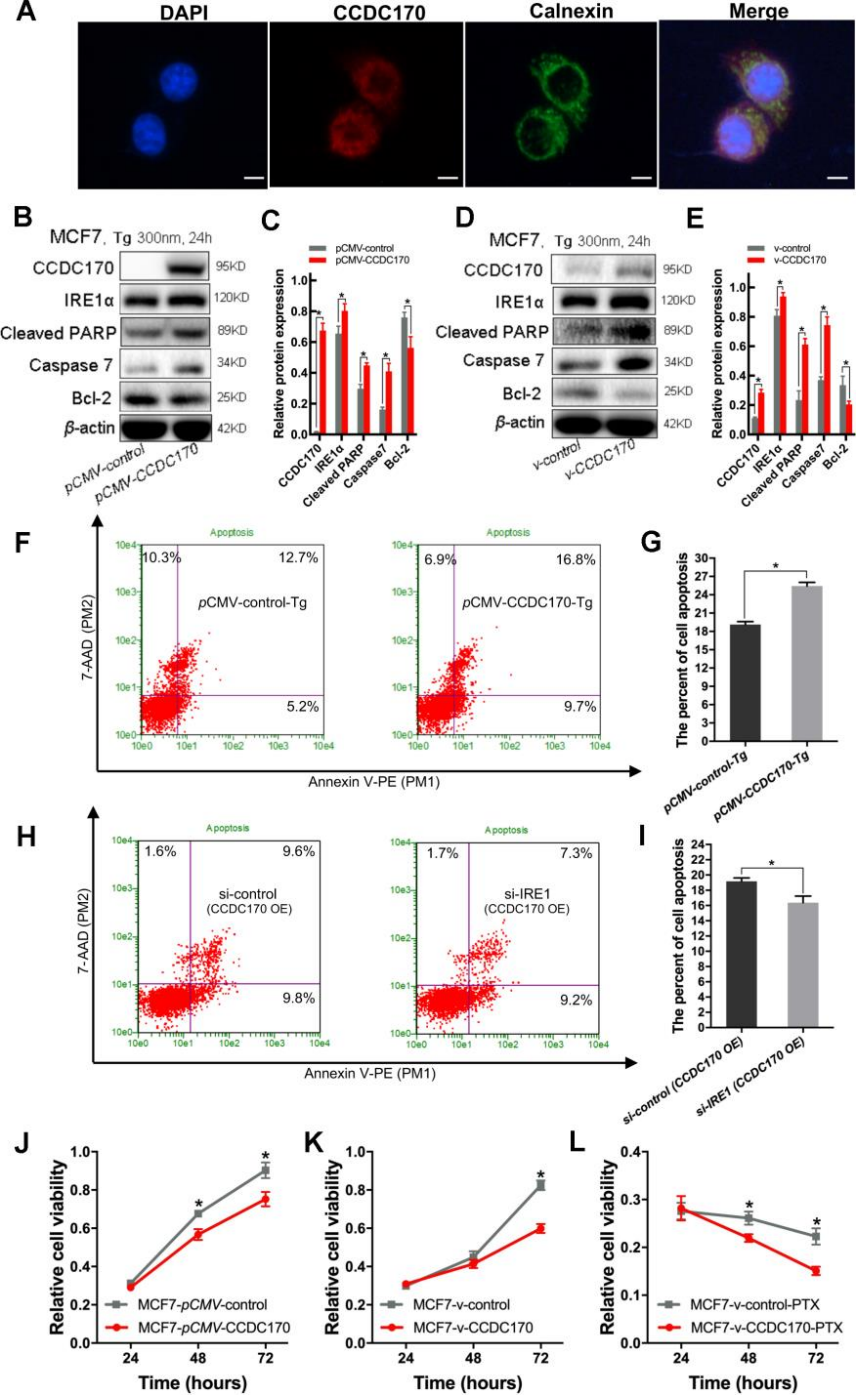
**CCDC170 promoted cell apoptosis under ER stress.** (**A**) IF showed that protein localization of CCDC70 overlapped Calnexin partially. Scale bar: 50μm. (**B, D**) Representative western blot bands of Cleaved PARP, Caspase7, Bcl-2 in MCF7 cells when CCDC170 up-regulated transiently (**C**) and stably (**E**) under TG treatment. pCMV-CCDC170(control) represented CCDC170-transiently-overexpressing MCF7 cells and controls. v-CCDC170(control) represented CCDC170-stably-overexpressing MCF7 cells and controls. *β*-actin was used as a reference for calculating the relative protein expression. TG: Thapsigargin (300nM). (**F**) Representative images of flow cytometry using Annexin V-FITC and PI staining, (**G**) Column bar graph showing an increased proportion of early and late apoptotic cells after CCDC170 overexpression in MCF7 cells treated with TG. 3000 cells were calculated. (**H**) Representative images of flow cytometry using Annexin V-FITC and PI staining, (**I**) Column bar graph showing a decreased proportion of early and late apoptotic cells after IRE1 knockdown in MCF7 cells with CCDC170 overexpression. 3000 cells were calculated. *CCDC170 OE*: MCF7 cells that transiently overexpressed CCDC170. Detection of cell viability via MTT assay. Transient (**J**) or stable (**K**) overexpression CCDC170, the growth of the cell was suppressed in MCF7 breast cancer cells. (**L**) The cell viability of CCDC170-stably-overexpressing MCF7 cells was significantly lower than that of control cells treated with PTX. PTX: paclitaxel (100nM). Each group was repeated at least three times. The error bars presented as mean ± Standard Error of Mean (SEM) with analysis of unpaired Student’s t-test. *P< 0.05, compared with the control group.

We then investigated the effects of CCDC170 on breast cancer cell apoptosis and viability. In cells that were transiently transfected with a eukaryotic CCDC170 expression vector for 24 hours, the percentage of apoptotic cells was 1.31 times higher than in the control group (*P =* 0.044; Figure 4A, 4B). On the other hand, when CCDC170 was transiently silenced using small interfering RNA (siRNA), flow cytometry revealed that the percentage of apoptotic cells decreased by 28.30% (*P =* 0.047; Figure 4C, 4D). We also found that the number of apoptotic nuclei increased in cells that transiently overexpressed CCDC170 (*P =* 7.00 ×10^-3^; Figure 4E, 4F). The expression of Caspase-12 increased when CCDC170 was upregulated transiently (*P =* 0.005; Figure 4G, 4H) or stably (*P =* 0.002; Figure 4I, 4J). Furthermore, whether CCDC170 was overexpressed transiently (*P =* 0.001; Figure 5J) or stably (*P =* 0.003; Figure 5K) in MCF7 breast cancer cells, the cell viability was significantly lower than that of the control group in a 3-(4,5-dimethylthiazol-2-yl)-2,5-diphenyltetrazolium bromide (MTT) assay.

Next, we used immunohistochemistry to assess the localization of CCDC170 in paraffin-embedded breast cancer tissues, and found that it was expressed in the cytoplasm. Immunofluorescence staining further indicated that CCDC170 partially overlapped with calnexin, a marker of the endoplasmic reticulum (Figure 5A). Nevertheless, no binding between CCDC170 and IRE1α was observed in a co-immunoprecipitation assay (data not presented).

Endoplasmic reticulum stress is ubiquitous in the tumor microenvironment; thus, we simulated endoplasmic reticulum stress *in vitro* by treating MCF7 cells with 300 nm thapsigargin for 24 hours. Western blot analyses demonstrated that MCF7 breast cancer cells that transiently overexpressed CCDC170 expressed higher levels of IRE1α (*P =* 0.018), cleaved poly [ADP-ribose] polymerase (PARP, an apoptotic regulatory protein; *P =* 0.001) and Caspase-7 (*P =* 0.001) and lower levels of Bcl-2 (an apoptosis-inhibiting protein; *P =* 0.013) than control cells under endoplasmic reticulum stress (Figure 5B, 5C). Likewise, in MCF7 cells that stably overexpressed CCDC170, the expression of IRE1α (*P =* 0.009), cleaved PARP (*P =* 9.27×10^-4^) and Caspase-7 (*P =* 4.36×10^-4^) increased while the expression of Bcl-2 decreased (*P =* 0.025) compared with control cells under endoplasmic reticulum stress for 24 hours (Figure 5D, 5E). Flow cytometry analysis demonstrated that the proportion of apoptotic cells was 1.37 times higher in the CCDC170-overexpressing group than in the control group after 24 hours of endoplasmic reticulum stress (*P =* 0.001; Figure 5F, 5G). In addition, we found that the percentage of apoptotic cells decreased when IRE1 was knocked down without endoplasmic reticulum stress in MCF7 cells that transiently overexpressed CCDC170 (*P* = 0.046; Figure 5H, 5I).

To compare the paclitaxel chemosensitivity between breast cancer cells with different CCDC170 levels, we treated CCDC170-stably-overexpressing MCF7 cells and control MCF7 cells with 100 nM paclitaxel. An MTT assay indicated that paclitaxel treatment inhibited growth more effectively in CCDC170-stably-overexpressing cells than in control cells (Figure 5L).

## DISCUSSION

Several single-nucleotide polymorphisms around human *CCDC170* have been identified as important breast cancer risk indicators in Chinese women [25–27]. In addition, *CCDC170* was found to be tightly co-expressed with *ESR1* in breast tumor biopsies and cells [6]. A previous study demonstrated that *CCDC170* was fused to *ESR1* and employed the constitutively active *ESR1* promoter to induce the expression of a truncated form of *CCDC170*. The fused gene was enriched in luminal B breast tumors and was found to promote a more aggressive phenotype by enhancing cell migration, invasion, anchorage-independent growth and endocrine therapy resistance [7]. However, it has been proposed that *CCDC170* can function as either an oncogene or a tumor suppressor [28]. Indeed, higher *CCDC170* expression has been associated with a better prognosis in certain breast cancer subtypes, but with a poorer prognosis in others [6, 29]. Though these data have highlighted the importance of the *CCDC170* gene and its fusion protein in breast cancer, the pathobiology and clinical relevance of CCDC170 have remained unclear.

Microarrays are a powerful technology for assessing expression profiles across entire genomes and discovering DEGs that contribute to the phenotypes, treatment responses and heterogeneity of complex diseases [30–34]. In the present work, we first used a gene chip to identify genes that were differentially expressed upon ectopic CCDC170 expression in MCF7 breast cancer cells. Bioinformatic analyses indicated that *IRE1* was the most notably elevated gene in the top 20 apoptotic pathways.

IRE1α is a stress sensor that performs a myriad of cellular functions in a complex signaling network [35–39]. IRE1α is well known to cleave *XBP1* in the most conserved arm of the unfolded protein response, which is an important determinant of cell death and survival [40]. IRE1α expression has been reported to be higher in breast cancer tissues than in the surrounding noncancerous tissues [41]. In the present study, the positive correlation between *CCDC170* and *IRE1* levels in our microarray analysis was validated at both the mRNA and protein levels in breast cancer tissues and cells.

XBP1s is a transcription factor that can induce the expression of *ESR1*, and these two genes are co-expressed in breast cancer tissues [13–16]. A recent study revealed that an *XBP1* gene signature was expressed at significantly higher levels in ERα+ or non-triple-negative breast cancer samples than in ERα- or triple-negative breast cancer samples, respectively [42]. Our immunohistochemical analyses demonstrated that CCDC170 and XBP1s were preferentially expressed in ERα+ breast cancer tissues, consistent with previous observations [6, 42]. Western blotting and immunofluorescence analyses indicated that ERα, IRE1α and XBP1s levels correlated with CCDC170 levels in MCF7 breast cancer cells. These findings suggested the existence of a potential regulatory loop among these proteins.

While the physiological function of CCDC170 has not been well understood, previous studies have suggested that *CCDC170* expression alters the prognosis of breast cancer patients. A study conducted in Japanese populations demonstrated that higher *CCDC170* expression correlated with better relapse-free survival in luminal A subtype patients, but correlated inversely with relapse-free survival in luminal B subtype patients [29]. Dunbier et al. reported that higher *CCDC170* expression prolonged the DFS of ERα+ patients [6]. Similar to the latter results, our survival analysis revealed that higher CCDC170 expression was associated with better DFS in the entire patient cohort, but was not associated with OS. In further analyses, we found that higher CCDC170 expression predicted significantly better DFS in the chemotherapy cohort and the radiation cohort, and tended (though not statistically significantly) to be associated with a better prognosis in the endocrine therapy cohort and the non-endocrine therapy cohort. We further analyzed the relevance of *CCDC170*, *IRE1* and *XBP1* expression to breast cancer prognosis in two independent cohorts (TCGA and GEO), and found that higher expression of *CCDC170*, *IRE1* and *XBP1* correlated with better OS in GEO. Combined with our initial observation that CCDC170 overexpression mainly induced genes involved in apoptosis, these results suggested that CCDC170 is a tumor inhibitor. Notably, although significant results were obtained in our breast cancer tissues, the sample size was relatively moderate. Though we reached consistent conclusions using data from TCGA and GEO, larger samples size will be needed to validate these findings in subsequent research.

In our functional analyses, we discovered that the overexpression of CCDC170 increased the apoptosis of MCF7 cells, while the silencing of CCDC170 reduced it. Remarkably, we found that Caspase-12 was induced in both transiently- and stably-CCDC170-overexpressing MCF7 cells. We also observed that either stable or transient CCDC170 overexpression clearly inhibited the growth of breast cancer cells. Caspase-12, which is located on the cytoplasmic side of the endoplasmic reticulum, is specifically activated during endoplasmic reticulum stress and is a key initiator of cell death [43, 44]. In tumor cells, endoplasmic reticulum stress is common because the endoplasmic reticular environment is disturbed [45]. We simulated endoplasmic reticulum stress *in vitro* by treating MCF7 cells for 24 hours with thapsigargin, which is well known to induce endoplasmic reticulum stress by inhibiting the Ca^2+^-ATPase pump [46]. CCDC170 more noticeably induced apoptosis (and upregulated IRE1α) in breast cancer cells under endoplasmic reticulum stress. Western blotting revealed that pro-apoptotic molecules such as cleaved PARP and Caspase-7 were upregulated in CCDC170-overexpressing cells compared with control cells under endoplasmic reticulum stress, while the anti-apoptotic protein Bcl-2 was downregulated. In addition, the rate of apoptosis was higher in CCDC170-overexpressing cells than in control cells under endoplasmic reticulum stress. However, the knockdown of IRE1α impaired the pro-apoptotic effects of CCDC170. Interestingly, although we found that CCDC170 was partly located on the endoplasmic reticulum membrane, we detected no direct binding between CCDC170 and IRE1α in our co-immunoprecipitation experiments. Thus, the effects of CCDC170 on breast cancer cells may partly depend on the biochemical activation of the IRE1α pathway

A previous study indicated that CCDC170 could bind to perinuclear microtubules, enhance their stability and suppress cell migration [28]. Paclitaxel, one of the taxanes, is an antineoplastic chemotherapeutic agent that stabilizes microtubules, and is frequently used to treat cancers such as breast cancer [47]. We found that CCDC170-overexpressing MCF7 cells were more sensitive than control cells to paclitaxel treatment. We also found CCDC170 overexpression increased the expression level of ERα. Although ERα is an important target of endocrine therapy, many proteins work together to maintain a functional niche environment. CCDC170 is also expected to become a therapeutic target in cancer.

The human cDNA microarray analysis in the present study has provided clues into the functions of CCDC170 in breast cancer. Our DEG analysis in MCF7 breast cancer cells revealed that *IRE1* was upregulated in CCDC170-overexpressing cells. Higher CCDC170 expression was associated with a better prognosis in breast cancer patients, and the expression of IRE1α, XBP1s and ERα correlated with the expression of CCDC170. The impact of CCDC170 on cell fate could be partially attributed to the IRE1α-XBP1s pathway. Thus, CCDC170 may have considerable potential as a therapeutic target for breast cancer. The broader involvement and clinical relevance of CCDC170 in the pathogenesis of breast cancer will be the focus of future investigations.

## MATERIALS AND METHODS

### Clinical samples

We obtained 100 formalin-fixed, paraffin-embedded tumor tissues from patients with breast invasive ductal carcinoma who had undergone surgical resection at Tianjin Medical University Cancer Institute and Hospital from December 2008 to May 2009. All the patients were followed up effectively through telephone calls or outpatient electronic medical records until September 2017. Detailed demographic data and clinicopathological information were collected retrospectively. The use of the patients’ specimens and information was approved by the ethics committee of Tianjin Medical University Cancer Institute and Hospital.

### Breast cancer data from public databases

We collected the gene expression profiles of 742 patients with stage I, II or III invasive ductal carcinoma from TCGA (http://www.cbioportal.org/index.do) and 3409 samples from GSE96058 in the GEO repository (https://www.ncbi.nlm.nih.gov/geo/) All gene expression data were uniformly normalized, and pathological information was downloaded. Details on the patients involved in our study are shown in Supplementary Tables 2, 3.

### Cell maintenance

MCF7 breast cancer cells were obtained from the American Type Culture Collection. The cells were maintained in RPMI-1640 medium (Invitrogen, USA) supplemented with 10% fetal bovine serum (Thermo Fisher Scientific, USA) at 37° C in a humidified atmosphere containing 5% CO_2_.

### Cell transformation

DH5α E. coli were transformed with plasmid DNA (either the pCMV-N-Flag-CCDC170 vector or the pCMV-N-Flag control vector) using the heat shock method. Specifically, 10 ng of the plasmid was mixed with 5 uL of DH5α E. coli and placed on ice for 30 min. Then, the mixture of chemically competent bacteria and DNA was incubated at 42° C for 90 s (heat shock) and placed back on ice for 10 min. Subsequently, 500 uL of lysogeny broth medium was added, and the mixture was incubated at 37° C for 0.5-1 hour. Then, the mixture was centrifuged for 5 min at 8000 x *g*, and the supernatant was removed. Freshly prepared agar plates with ampicillin were inoculated with 150 uL of this mixture overnight at 37° C.

Subsequently, 4-6 mL of lysogeny broth medium was inoculated with single colonies picked from the freshly prepared agar plates and incubated on a shaker at 37° C for 12-16 hours. The plasmid was extracted and purified using the EZgene™ EndoFree Plasmid ezFlow Miniprep Kit II (PD1222, BIOMIGA) in strict adherence to the manufacturer’s instructions. The plasmid concentration was measured on a NanoDrop 2000c Spectrophotometer (Thermo Fisher Scientific, Waltham, MA, USA).

### Transfection

MCF7 cells (3 × 10^5^) were seeded on six-well plates for 12-16 hours. For the transient CCDC170 overexpression experiments, the cells were transfected with the pCMV-N-Flag-CCDC170 or pCMV-N-Flag control vector using Lipofectamine^TM^ 3000 Reagent (Invitrogen, USA). For the CCDC170 and IRE1 knockdown experiments, the cells were transfected with CCDC170 siRNA, IRE1 siRNA or the corresponding negative control (NC) siRNA (GenePharma, China) using Lipofectamine^TM^ RNAiMAX transfection reagent (Invitrogen, USA) according to the manufacturer’s instructions.

The CCDC170 siRNAs targeted the following sequences:

siRNA-1: sense 5′-GCCCACAAUUUGCAGAGAATT-3′

antisense 5′-UUCUCUGCAAAUUGUGGGCTT-3′

siRNA-2: sense 5′-GCAGCAACUUUGGUCAAAUTT-3′

antisense 5′-AUUUGACCAAAGUUGCUGCTT-3′

NC: sense 5′-UUCUCCGAACGUGUCACGUTT-3′

antisense 5′-ACGUGACACGUUCGGAGAATT-3′

The IRE1 siRNAs targeted the following sequences:

siRNA-1: sense 5′-GCAGAUAGUCUCUGCCCAUTT-3′

antisense 5′-AUGGGCAGAGACUAUCUGCTT-3′

siRNA-2: sense 5′-GCAAGAACAAGCUCAACUATT-3′

antisense 5′-UAGUUGAGCUUGCUUGCTT-3′

NC: sense 5′-UUCUCCGAACGUGUCACGUTT-3′

antisense 5′-ACGUGACACGUUCGGAGAATT-3′

### Lentiviral infection

CCDC170 was stably overexpressed using the recombinant lentiviral vector LV8(EF-1a/RFP+Puro) (Shanghai GeneChem Co., Ltd., Cina), and the empty vector was used as a negative control. MCF7 cells were plated on six-well dishes at 30-40% confluence and infected with the retroviruses. Polybrene was added at a concentration of 5 μg/mL to enhance the infection efficiency. Seventy-two hours after the infection, 2 μg/mL puromycin was applied for 10 days to select the stable pooled cell population.

### RNA extraction, amplification and labeling

Total RNA was extracted using an RNeasy Mini Kit (Qiagen, Valencia, CA, USA) in accordance with the manufacturer’s instructions. The overall RNA concentration and purity were measured on a NanoDrop 2000 Spectrophotometer. Total RNA was amplified and labeled using a Low Input Quick Amp Labeling Kit (Agilent Technologies, USA).

### Microarray construction

The effects of CCDC170 on mRNA expression in MCF7 cells were analyzed using microarrays. After transcription, purification and fragmentation, samples were hybridized onto Agilent Whole Human Genome 4×44K 60 mer oligonucleotide arrays at 60° C for 16 hours. The arrays were then washed and scanned so that gene expression could be quantified. The fluorescence scanning image signals from the chip were converted to digital signals using Feature Extraction software v.10.7 (Agilent Technologies, USA).

### DEG identification

Raw microarray data were normalized using the Quantile algorithm after background correction. Each gene expression value was calculated as the weighted average of all the forward or reverse probe sets. The ratio of the signal intensity in the experimental group (with 24- or 48-hour CCDC170 overexpression) to that in the control group was used to analyze the chip data for each gene (fold ratio = experimental group/control group). The DEGs between the CCDC170-overexpressing and control MCF7 cells were selected according to the cut-off criteria of log|(fold ratio)| > 0.1761 and *P* < 0.05.

### Pathway enrichment analysis

The Kyoto Encyclopedia of Genes and Genomes database was used for the pathway enrichment analysis of the DEGs, including Annotation, Visualization and Integrated Discovery. The data were analyzed using the “clusterProfiler” package in R, and *P* < 0.05 was considered statistically significant.

### Immunohistochemistry

Formalin-fixed, paraffin-embedded tissue sections were stained at 4° C overnight using the following antibodies: 1:100 anti-CCDC170 (PA5-34723, Thermo Fisher Scientific, USA), 1:400 anti-XBP1 (PA5-27650, Thermo Fisher Scientific, USA) and 1:50 anti-IRE1α (H-190, Santa Cruz Biotechnology, USA). For antigen retrieval, the tissue sections were boiled in a sodium citrate solution (pH 6.0) in a pressure cooker for 2.5 min. For antibody visualization, an Ultra View DAB Detection kit (Dako, 20015510) was applied. Negative control samples were treated in the same manner without the addition of primary antibodies.

Comprehensive staining scores were calculated based on the staining intensity and percentage of positive cells in five randomly chosen visual fields. The staining intensity was scored as follows: 0 (no staining), 1 (weak), 2 (moderate) and 3 (strong). The mean percentage of positive cells was scored as follows: 0 (<5%), 1 (5-25%), 2 (26-50%), 3 (51-75%) and 4 (76-100%). The final scores were calculated as the staining proportion score multiplied by the staining intensity score. Based on their final scores, patients were divided into the low (immunohistochemistry scores ≤ 2) and high (immunohistochemistry scores > 2) expression groups.

### Western blotting

Total proteins were electrophoretically separated on sodium dodecyl sulfate polyacrylamide gels (10-12%) according to the molecular size of the target protein, and were subsequently transferred onto polyvinylidene difluoride membranes. After being blocked with 5% skim milk, the membranes were incubated at 4° C overnight with the following primary antibodies: anti-CCDC170 (1:500, PA5-34723, Thermo Fisher Scientific, USA), anti-XBP1 (1:1000, PA5-27650, Thermo Fisher Scientific, USA), anti-IRE1α (1:1000, 14C10, Cell Signaling Technology, USA), anti-cleaved PARP (1:1000, 9542S, Cell Signaling Technology), anti-Caspase-12 (1:500, sc-21747, Santa Cruz Biotechnology, USA), anti-Caspase-7 (1:1000, D2Q3L, Cell Signaling Technology, USA), anti-Bcl2 (1:500, ab692, Abcam, USA) and anti-*β*-actin (1:2000, Santa Cruz Biotechnology, USA). Then, the membranes were washed thoroughly and incubated with secondary antibodies (1:3000 anti-mouse or 1:5000 anti-rabbit) at room temperature for 1 hour. The signals were visualized using the enhanced chemiluminescence method (Immobilon Western Chemiluminescent HRP Substrate, Millipore, USA). The samples were analyzed in duplicate, and the experiment was performed three times.

### Immunofluorescence

Cells were grown on a microscope cover glass (PA 15275, Thermo Fisher Scientific, USA) laid on the bottom of the well of a 24-well plate (1 × 10^4^ cells per well). The cells were fixed with 4% paraformaldehyde for 15 min, permeabilized with 0.2% Triton X-100 for 20 min and blocked with 10% goat serum for 30 min at room temperature. Then, the slides were incubated with anti-CCDC170 (1:200, PA5-34723, Thermo Fisher Scientific, USA) or anti-calnexin (1:500, MA3-027, Invitrogen, USA) primary antibodies overnight at 4° C. The slides were subsequently incubated for 1 hour at room temperature with fluorescence-conjugated secondary antibodies (A21206/Alexa Fluor®488 donkey anti-rabbit IgG or A21203/Alexa Fluor®594 donkey anti-mouse IgG) diluted 1:1000. The nuclei were counterstained with 4’,6-diamidino-2-phenylindole (DAPI) after the slides had been washed thoroughly. Images were captured using phase-contrast fluorescence microscopy.

### Flow cytometric apoptosis detection

The percentage of apoptotic cells was ascertained using flow cytometry, based on the binding of Annexin V-PE and 7-ADD. Samples of 2x10^4^ to 2x10^5^ cells were prepared in 10% bovine serum albumin, according to the manufacturer’s instructions. Then, 100 uL of Guava Nexin® Reagent (4500-0450, Millipore, USA) was added to each sample, and the mixture was incubated for 20 min at room temperature in the dark. Finally, the samples were analyzed on a Guava system. The samples were analyzed in duplicate, and the experiment was performed three times.

### Terminal deoxynucleotidyl transferase dUTP nick end labeling (TUNEL) assay

Late-stage apoptosis was detected with a TUNEL BrightGreen Apoptosis Detection Kit (Vazyme Biotech, A112-03). Adherent MCF7 cells were cultured on Chamber Slides (PA 15275, Thermo Fisher Scientific, USA) in 24-well plates at a density of 1 × 10^4^ cells per well. The cells were then fixed, washed, labeled and detected in accordance with the kit manual. After the TUNEL labeling, the nuclei were labeled with DAPI. Fluorescein isothiocyanate-12-dUTP-labeled DNA was observed directly under a fluorescence microscope. For the positive control slide, cells were permeabilized and treated with DNase I. For the negative control, no terminal deoxynucleotidyl transferase enzyme was added.

### MTT assay

The MTT assay involves the conversion of the water-soluble yellow dye MTT to insoluble purple formazan by the action of mitochondrial reductase. MCF7 cells were reseeded in a sterile 96-well plate at a density of 3000 cells per well and grown for three days. Then, 50 μL of diluted MTT was added to each well and incubated with the cells for 4 hours in an incubator. The medium was removed, and 100 μL of dimethyl sulfoxide was added to each well to dissolve the formazan. Finally, the optical density of each well was measured on a spectrophotometer at a wavelength of 595 nm. The samples were analyzed in triplicate, and the experiment was repeated three times.

### Statistical analyses

Statistical analyses were performed with SPSS 19.0 (SPSS Inc., Chicago, IL, USA). Categorical variables were compared using the Chi-square test, while continuous variables were analyzed using nonparametric tests (Kruskal-Wallis test and Mann-Whitney test), one-way analysis of variance (ANOVA) and Student’s t-test. Spearman correlations were calculated because the data were not normally distributed, even after log-transformation.

The survival time was measured in months from the date of breast cancer diagnosis until recurrence, metastasis or death. OS was evaluated from the date of diagnosis to the date of death or last follow-up. DFS was calculated as the time from diagnosis to the observation of disease progression or death for any reason. Kaplan-Meier analyses and log-rank tests were performed to estimate the probability of OS and DFS. Cox logistic regression models with 95% CIs were used to evaluate the independent prognostic factors.

Data from cell experiments were analyzed using unpaired Student’s t-tests, and images based on the statistical analyses were made in GraphPad Prism 5.0 (GraphPad Software Inc, La Jolla, CA, USA). All hypothetical tests were two-sided, and *P*-values less than 0.05 were considered statistically significant in all tests.

### Ethics approval and consent to participate

Permission to use the paraffin-embedded tissues from this study for research purposes was provided by the Department of Breast Pathology, Tianjin Medical University Cancer Institute and Hospital. The ethics committee of Tianjin Medical University approved the study.

### Availability of data and materials

The gene expression profiles used in the current study are available on the websites of TCGA (www.cbioportal.org/index.do) and GEO (www.ncbi.nlm.nih.gov/geo; accession number GSE96058).

## Supplementary Material

Supplementary Figures

Supplementary Tables

## References

[1] Bray F, Ferlay J, Soerjomataram I, Siegel RL, Torre LA, Jemal A. Global cancer statistics 2018: GLOBOCAN estimates of incidence and mortality worldwide for 36 cancers in 185 countries. CA Cancer J Clin. 2018; 68:394–424. 10.3322/caac.21492 30207593

[2] Mahdavi M, Nassiri M, Kooshyar MM, Vakili-Azghandi M, Avan A, Sandry R, Pillai S, Lam AK, Gopalan V. Hereditary breast cancer; genetic penetrance and current status with BRCA. J Cell Physiol. 2019; 234:5741–50. 10.1002/jcp.27464 30552672

[3] Zehr KR. Sporadic and hereditary breast cancer genetics. Radiol Technol. 2018; 90:51M–64M. 30352929

[4] Dunning AM, Healey CS, Pharoah PD, Teare MD, Ponder BA, Easton DF. A systematic review of genetic polymorphisms and breast cancer risk. Cancer Epidemiol Biomarkers Prev. 1999; 8:843–54. 10548311

[5] Peng S, Lü B, Ruan W, Zhu Y, Sheng H, Lai M. Genetic polymorphisms and breast cancer risk: evidence from meta-analyses, pooled analyses, and genome-wide association studies. Breast Cancer Res Treat. 2011; 127:309–24. 10.1007/s10549-011-1459-5 21445572

[6] Dunbier AK, Anderson H, Ghazoui Z, Lopez-Knowles E, Pancholi S, Ribas R, Drury S, Sidhu K, Leary A, Martin LA, Dowsett M. ESR1 is co-expressed with closely adjacent uncharacterised genes spanning a breast cancer susceptibility locus at 6q25.1. PLoS Genet. 2011; 7:e1001382. 10.1371/journal.pgen.1001382 21552322 PMC3084198

[7] Veeraraghavan J, Tan Y, Cao XX, Kim JA, Wang X, Chamness GC, Maiti SN, Cooper LJ, Edwards DP, Contreras A, Hilsenbeck SG, Chang EC, Schiff R, Wang XS. Recurrent ESR1-CCDC170 rearrangements in an aggressive subset of oestrogen receptor-positive breast cancers. Nat Commun. 2014; 5:4577. 10.1038/ncomms5577 25099679 PMC4130357

[8] Bardou VJ, Arpino G, Elledge RM, Osborne CK, Clark GM. Progesterone receptor status significantly improves outcome prediction over estrogen receptor status alone for adjuvant endocrine therapy in two large breast cancer databases. J Clin Oncol. 2003; 21:1973–79. 10.1200/JCO.2003.09.099 12743151

[9] Liu CY, Xu Z, Kaufman RJ. Structure and intermolecular interactions of the luminal dimerization domain of human IRE1alpha. J Biol Chem. 2003; 278:17680–87. 10.1074/jbc.M300418200 12637535

[10] Glembotski CC. Endoplasmic reticulum stress in the heart. Circ Res. 2007; 101:975–84. 10.1161/CIRCRESAHA.107.161273 17991891

[11] Uemura A, Oku M, Mori K, Yoshida H. Unconventional splicing of XBP1 mRNA occurs in the cytoplasm during the mammalian unfolded protein response. J Cell Sci. 2009; 122:2877–86. 10.1242/jcs.040584 19622636

[12] Hetz C, Papa FR. The Unfolded Protein Response and Cell Fate Control. Mol Cell. 2018; 69:169–181. 10.1016/j.molcel.2017.06.017 29107536

[13] Andres SA, Wittliff JL. Relationships of ESR1 and XBP1 expression in human breast carcinoma and stromal cells isolated by laser capture microdissection compared to intact breast cancer tissue. Endocrine. 2011; 40:212–21. 10.1007/s12020-011-9522-x 21858728

[14] Andres SA, Wittliff JL. Co-expression of genes with estrogen receptor-α and progesterone receptor in human breast carcinoma tissue. Horm Mol Biol Clin Investig. 2012; 12:377–90. 10.1515/hmbci-2012-0025 25436697

[15] Fang Y, Yan J, Ding L, Liu Y, Zhu J, Huang C, Zhao H, Lu Q, Zhang X, Yang X, Ye Q. XBP-1 increases ERalpha transcriptional activity through regulation of large-scale chromatin unfolding. Biochem Biophys Res Commun. 2004; 323:269–74. 10.1016/j.bbrc.2004.08.100 15351732

[16] Ding L, Yan J, Zhu J, Zhong H, Lu Q, Wang Z, Huang C, Ye Q. Ligand-independent activation of estrogen receptor alpha by XBP-1. Nucleic Acids Res. 2003; 31:5266–74. 10.1093/nar/gkg731 12954762 PMC203316

[17] Mortezaee K, Najafi M, Farhood B, Ahmadi A, Potes Y, Shabeeb D, Musa AE. Modulation of apoptosis by melatonin for improving cancer treatment efficiency: an updated review. Life Sci. 2019; 228:228–41. 10.1016/j.lfs.2019.05.009 31077716

[18] Chalmers F, Mogre S, Son J, Blazanin N, Glick AB. The multiple roles of the unfolded protein response regulator IRE1α in cancer. Mol Carcinog. 2019; 58:1623–30. 10.1002/mc.23031 31041814 PMC6692187

[19] Chen L, Li Q, She T, Li H, Yue Y, Gao S, Yan T, Liu S, Ma J, Wang Y. IRE1α-XBP1 signaling pathway, a potential therapeutic target in multiple myeloma. Leuk Res. 2016; 49:7–12. 10.1016/j.leukres.2016.07.006 27518808

[20] Mimura N, Fulciniti M, Gorgun G, Tai YT, Cirstea D, Santo L, Hu Y, Fabre C, Minami J, Ohguchi H, Kiziltepe T, Ikeda H, Kawano Y, et al. Blockade of XBP1 splicing by inhibition of IRE1α is a promising therapeutic option in multiple myeloma. Blood. 2012; 119:5772–81. 10.1182/blood-2011-07-366633 22538852 PMC3382937

[21] Logue SE, McGrath EP, Cleary P, Greene S, Mnich K, Almanza A, Chevet E, Dwyer RM, Oommen A, Legembre P, Godey F, Madden EC, Leuzzi B, et al. Inhibition of IRE1 RNase activity modulates the tumor cell secretome and enhances response to chemotherapy. Nat Commun. 2018; 9:3267. 10.1038/s41467-018-05763-8 30111846 PMC6093931

[22] Ming J, Ruan S, Wang M, Ye D, Fan N, Meng Q, Tian B, Huang T. A novel chemical, STF-083010, reverses tamoxifen-related drug resistance in breast cancer by inhibiting IRE1/XBP1. Oncotarget. 2015; 6:40692–703. 10.18632/oncotarget.5827 26517687 PMC4747362

[23] Hu R, Warri A, Jin L, Zwart A, Riggins RB, Fang HB, Clarke R. NF-κB signaling is required for XBP1 (unspliced and spliced)-mediated effects on antiestrogen responsiveness and cell fate decisions in breast cancer. Mol Cell Biol. 2015; 35:379–90. 10.1128/MCB.00847-14 25368386 PMC4272419

[24] Sisinni L, Pietrafesa M, Lepore S, Maddalena F, Condelli V, Esposito F, Landriscina M. Endoplasmic reticulum stress and unfolded protein response in breast cancer: the balance between apoptosis and autophagy and its role in drug resistance. Int J Mol Sci. 2019; 20:857. 10.3390/ijms20040857 30781465 PMC6412864

[25] Zheng W, Long J, Gao YT, Li C, Zheng Y, Xiang YB, Wen W, Levy S, Deming SL, Haines JL, Gu K, Fair AM, Cai Q, et al. Genome-wide association study identifies a new breast cancer susceptibility locus at 6q25.1. Nat Genet. 2009; 41:324–28. 10.1038/ng.318 19219042 PMC2754845

[26] Wang Y, He Y, Qin Z, Jiang Y, Jin G, Ma H, Dai J, Chen J, Hu Z, Guan X, Shen H. Evaluation of functional genetic variants at 6q25.1 and risk of breast cancer in a Chinese population. Breast Cancer Res. 2014; 16:422. 10.1186/s13058-014-0422-x 25116933 PMC4303231

[27] Zhou L, He N, Feng T, Geng T, Jin T, Chen C. Association of five single nucleotide polymorphisms at 6q25.1 with breast cancer risk in northwestern China. Am J Cancer Res. 2015; 5:2467–75. 26396922 PMC4568782

[28] Jiang P, Li Y, Poleshko A, Medvedeva V, Baulina N, Zhang Y, Zhou Y, Slater CM, Pellegrin T, Wasserman J, Lindy M, Efimov A, Daly M, et al. The protein encoded by the CCDC170 breast cancer gene functions to organize the golgi-microtubule network. EBioMedicine. 2017; 22:28–43. 10.1016/j.ebiom.2017.06.024 28687497 PMC5552109

[29] Yamamoto-Ibusuki M, Yamamoto Y, Fujiwara S, Sueta A, Yamamoto S, Hayashi M, Tomiguchi M, Takeshita T, Iwase H. C6ORF97-ESR1 breast cancer susceptibility locus: influence on progression and survival in breast cancer patients. Eur J Hum Genet. 2015; 23:949–56. 10.1038/ejhg.2014.219 25370037 PMC4463506

[30] Tangir J, Bonafé N, Gilmore-Hebert M, Henegariu O, Chambers SK. SGK1, a potential regulator of c-fms related breast cancer aggressiveness. Clin Exp Metastasis. 2004; 21:477–83. 10.1007/s10585-004-4226-8 15679045

[31] Guo S, Russo IH, Lareef MH, Russo J. Effect of human chorionic gonadotropin in the gene expression profile of MCF-7 cells. Int J Oncol. 2004; 24:399–407. 14719117

[32] van‘t Veer LJ, Dai H, van de Vijver MJ, He YD, Hart AA, Mao M, Peterse HL, van der Kooy K, Marton MJ, Witteveen AT, Schreiber GJ, Kerkhoven RM, Roberts C, et al. Gene expression profiling predicts clinical outcome of breast cancer. Nature. 2002; 415:530–36. 10.1038/415530a 11823860

[33] van de Vijver MJ, He YD, van’t Veer LJ, Dai H, Hart AA, Voskuil DW, Schreiber GJ, Peterse JL, Roberts C, Marton MJ, Parrish M, Atsma D, Witteveen A, et al. A gene-expression signature as a predictor of survival in breast cancer. N Engl J Med. 2002; 347:1999–2009. 10.1056/NEJMoa021967 12490681

[34] Zheng T, Wang A, Hu D, Wang Y. Molecular mechanisms of breast cancer metastasis by gene expression profile analysis. Mol Med Rep. 2017; 16:4671–4677. 10.3892/mmr.2017.7157 28791367 PMC5647040

[35] Jin C, Jin Z, Chen NZ, Lu M, Liu CB, Hu WL, Zheng CG. Activation of IRE1α-XBP1 pathway induces cell proliferation and invasion in colorectal carcinoma. Biochem Biophys Res Commun. 2016; 470:75–81. 10.1016/j.bbrc.2015.12.119 26742428

[36] Cuevas EP, Eraso P, Mazón MJ, Santos V, Moreno-Bueno G, Cano A, Portillo F. LOXL2 drives epithelial-mesenchymal transition via activation of IRE1-XBP1 signalling pathway. Sci Rep. 2017; 7:44988. 10.1038/srep44988 28332555 PMC5362953

[37] Drogat B, Auguste P, Nguyen DT, Bouchecareilh M, Pineau R, Nalbantoglu J, Kaufman RJ, Chevet E, Bikfalvi A, Moenner M. IRE1 signaling is essential for ischemia-induced vascular endothelial growth factor-a expression and contributes to angiogenesis and tumor growth in vivo. Cancer Res. 2007; 67:6700–07. 10.1158/0008-5472.CAN-06-3235 17638880

[38] Li XX, Zhang HS, Xu YM, Zhang RJ, Chen Y, Fan L, Qin YQ, Liu Y, Li M, Fang J. Knockdown of IRE1α inhibits colonic tumorigenesis through decreasing β-catenin and IRE1α targeting suppresses colon cancer cells. Oncogene. 2017; 36:6738–46. 10.1038/onc.2017.284 28825721

[39] Lhomond S, Avril T, Dejeans N, Voutetakis K, Doultsinos D, McMahon M, Pineau R, Obacz J, Papadodima O, Jouan F, Bourien H, Logotheti M, Jégou G, et al. Dual IRE1 RNase functions dictate glioblastoma development. EMBO Mol Med. 2018; 10:e7929. 10.15252/emmm.201707929 29311133 PMC5840541

[40] Maurel M, Chevet E, Tavernier J, Gerlo S. Getting RIDD of RNA: IRE1 in cell fate regulation. Trends Biochem Sci. 2014; 39:245–54. 10.1016/j.tibs.2014.02.008 24657016

[41] Li C, Fan Q, Quan H, Nie M, Luo Y, Wang L. The three branches of the unfolded protein response exhibit differential significance in breast cancer growth and stemness. Exp Cell Res. 2018; 367:170–85. 10.1016/j.yexcr.2018.03.033 29601799

[42] Jiang D, Turner B, Song J, Li R, Diehn M, Le QT, Khatri P, Koong AC. Comprehensive analysis of the unfolded protein response in breast cancer subtypes. JCO Precis Oncol. 2017; 2017:PO.16.00073. 10.1200/PO.16.00073 29888341 PMC5992919

[43] Nakagawa T, Zhu H, Morishima N, Li E, Xu J, Yankner BA, Yuan J. Caspase-12 mediates endoplasmic-reticulum-specific apoptosis and cytotoxicity by amyloid-beta. Nature. 2000; 403:98–103. 10.1038/47513 10638761

[44] Szegezdi E, Fitzgerald U, Samali A. Caspase-12 and ER-stress-mediated apoptosis: the story so far. Ann N Y Acad Sci. 2003; 1010:186–94. 10.1196/annals.1299.032 15033718

[45] Yadav RK, Chae SW, Kim HR, Chae HJ. Endoplasmic reticulum stress and cancer. J Cancer Prev. 2014; 19:75–88. 10.15430/JCP.2014.19.2.75 25337575 PMC4204165

[46] Abdullahi A, Stanojcic M, Parousis A, Patsouris D, Jeschke MG. Modeling acute ER stress in vivo and in vitro. Shock. 2017; 47:506–13. 10.1097/SHK.0000000000000759 27755507 PMC5348263

[47] Abu Samaan TM, Samec M, Liskova A, Kubatka P, Büsselberg D. Paclitaxel’s mechanistic and clinical effects on breast cancer. Biomolecules. 2019; 9:789. 10.3390/biom9120789 31783552 PMC6995578

