# Supplementary material for: CCDC170 affects breast cancer apoptosis through IRE1 pathway
Source: Aging (Albany NY). 2020 Dec 3;13(1):1332–56. doi: 10.18632/aging.202315 (PMC7835043; doi:10.18632/aging.202315)
Supplement: Supplementary Figures [file aging-13-202315-s001.pdf]

SUPPLEMENTARY FIGURES

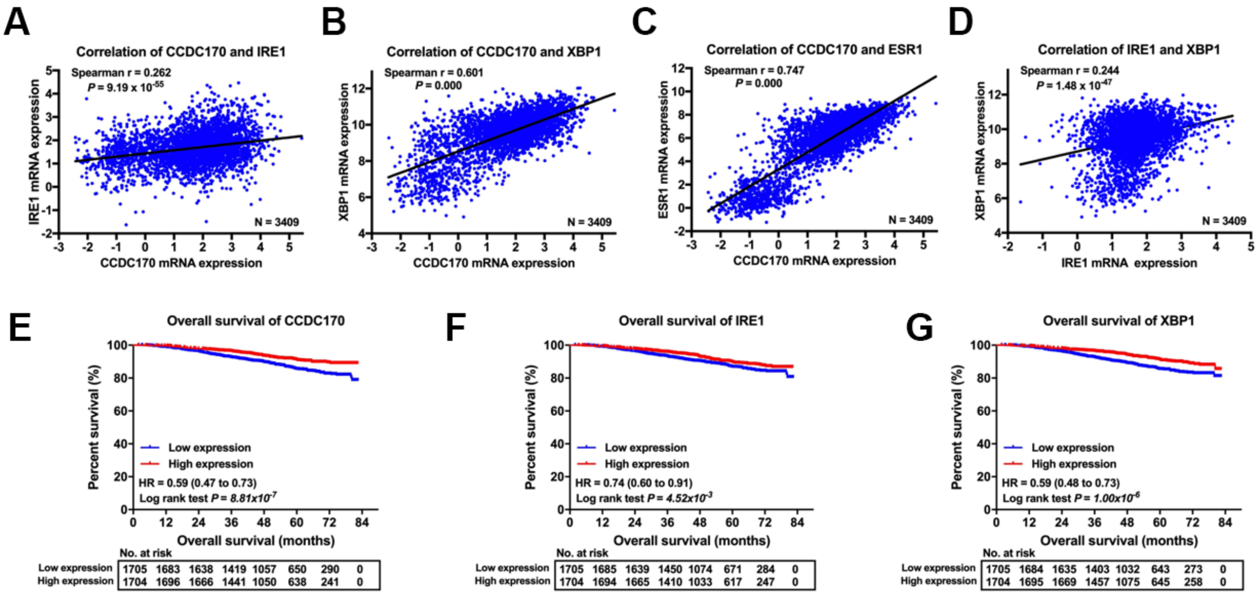

Supplementary Figure 1. Strong correlations between CCDC170, IRE1, XBP1 and ESR1 levels in breast cancer patients and their prognosis value. (**A**) The positive relationship between *CCDC170* and *IRE1*. (**B**) The positive correlation between *CCDC170* and *XBP1*. (**C**) The positive relationship between *CCDC170* and *ESR1*. (**D**) The positive relationship between *IRE1* and *XBP1*. High-expression groups for *CCDC170* (**E**), *IRE1* (**F**), and *XBP1* (**G**) exhibited better overall survival than their respective low-expression groups (based on the median). Datasets from GEO (GSE96058) were implemented for the correlation and survival analysis.

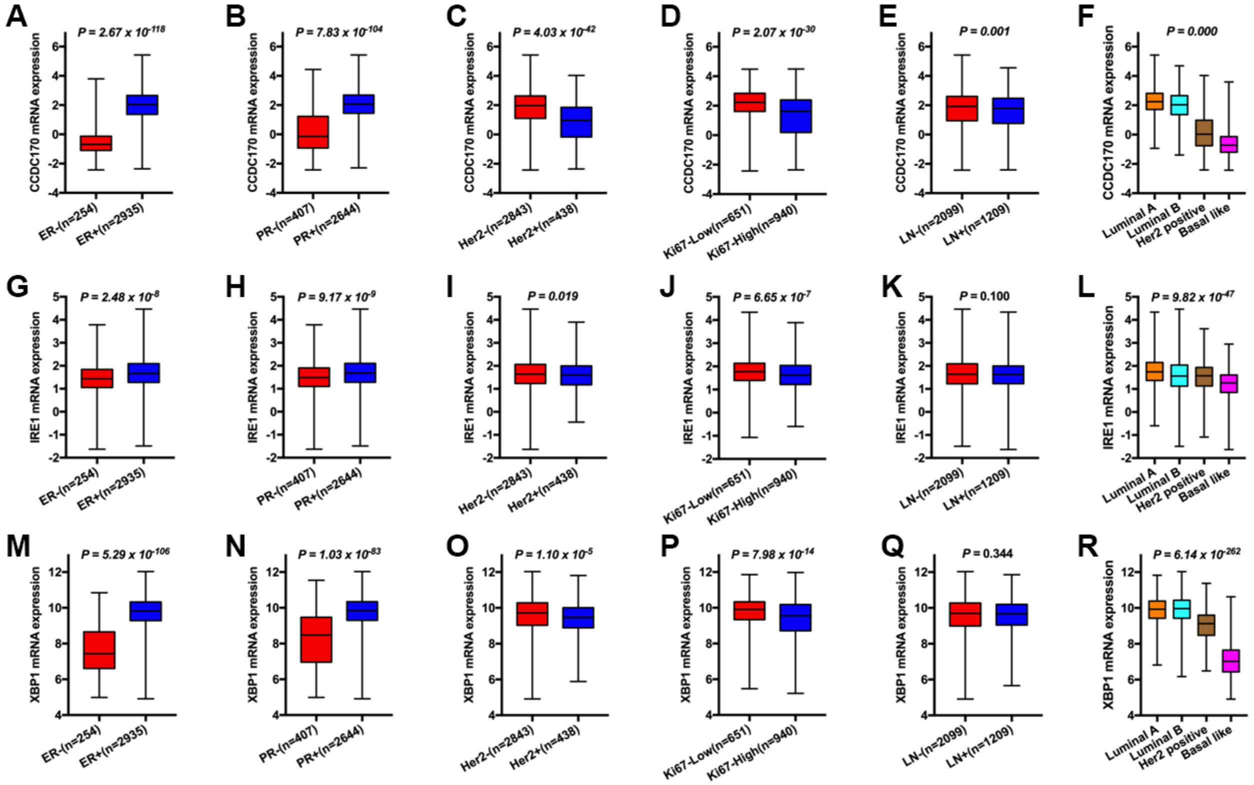

Supplementary Figure 2. The distribution of CCDC170, IRE1 and XBP1 gene signatures in different clinicopathological characteristics. (**A**, **G**, **M**) Differential distribution of *CCDC170, IRE1, XBP1* in the ER- group versus ER+ group. (**B**, **H**, **N**) Differential distribution of *CCDC170, IRE1, XBP1* in the PR- group versus PR+ group. (**C**, **I**, **O**) Differential distribution of *CCDC170, IRE1, XBP1* in the Her2- group versus Her2+ group. (**D**, **J**, **P**) Differential distribution of *CCDC170, IRE1, XBP1* in the Ki67-Low group versus Ki67-High group. (**E**, **K**, **Q**) Differential distribution of *CCDC170, IRE1, XBP1* in the LN- group versus LN+ group. (**F**, **L**, **R**) Differential distribution of *CCDC170, IRE1, XBP1* in the breast cancer groups with different Pam50 subtypes. The GSE96058 datasets were implemented for analysis. ER, estrogen receptor α; PR, progesterone receptor; Her2, human epidermal growth factor receptor 2; -, negative; +, positive; LN, lymph node status. The error bars presented as mean ± Standard Error of Mean (SEM) with analysis of Kruskal-Wallis test and Mann-Whitney test. P < 0.05 (two-tailed) was considered as statistically significant.

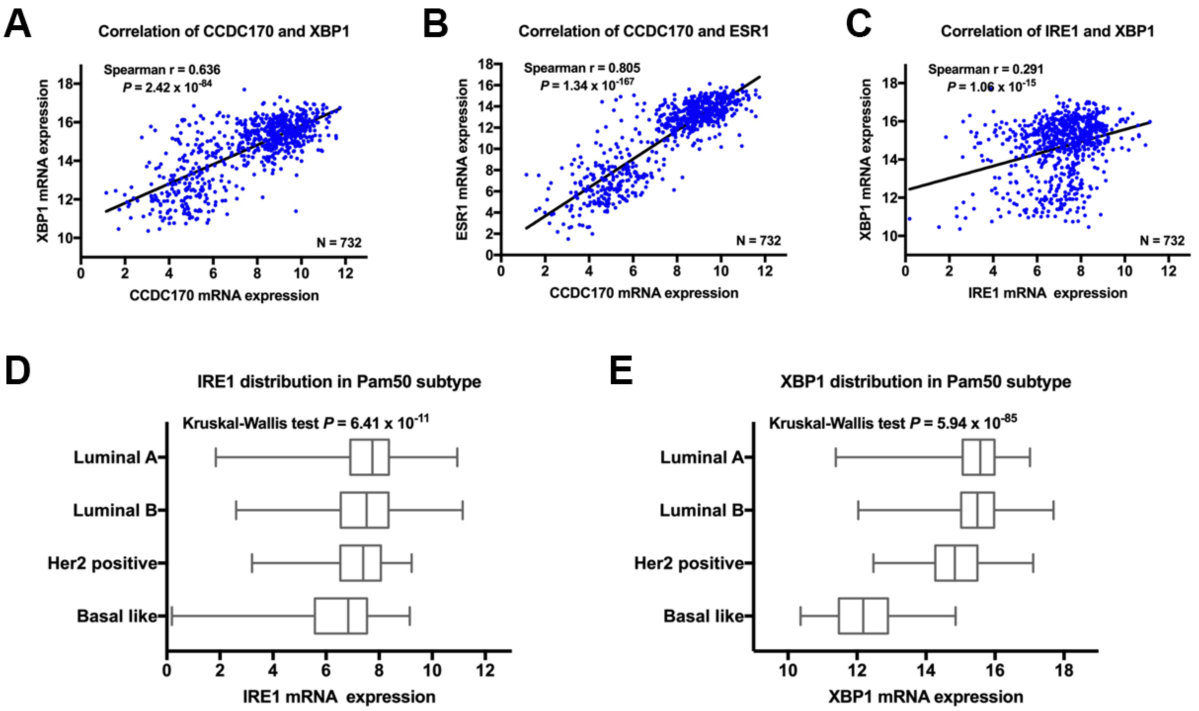

Supplementary Figure 3. The clinical significance of CCDC170, IRE1, and XBP1. (**A**) The positive relationship between *CCDC170* and *XBP1*. (**B**) The positive correlation between *CCDC170* and *ESR1*. (**C**) The positive relationship between *IRE1* and *XBP1*. (**D**) Differential distribution of *IRE1* mRNA expression in Pam50 subtypes. (**E**) Differential distribution of *XBP1* mRNA expression in Pam50 subtypes. Datasets from TCGA were implemented for correlation and distribution analysis.

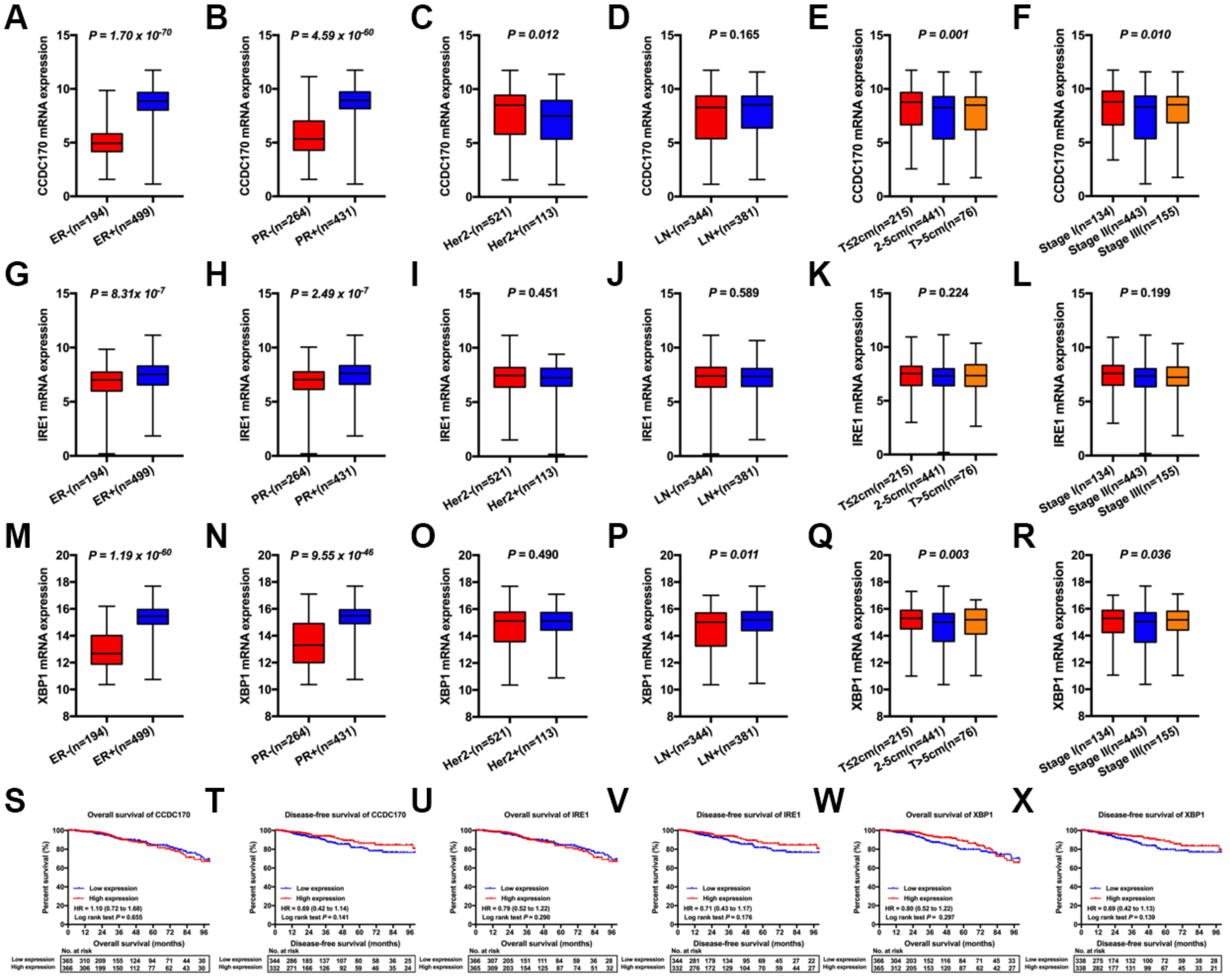

Supplementary Figure 4. The differential distribution of CCDC170, IRE1, XBP1 in breast cancer patients with different clinicopathological characteristics and their clinical significance. (**A**, **G**, **M**) Differential distribution of *CCDC170, IRE1, XBP1* in the ER- group versus ER+ group. (**B**, **H**, **N**) Differential distribution of *CCDC170, IRE1, XBP1* in the PR- group versus PR+ group. (**C**, **I**, **O**) Differential distribution of *CCDC170, IRE1, XBP1* in the Her2- group versus Her2+ group. (**D**, **J**, **P**) Differential distribution of *CCDC170, IRE1, XBP1* in the LN- group versus LN+ group. (**E**, **K**, **Q**) Differential distribution of *CCDC170, IRE1, XBP1* in the breast cancer groups with different tumor size. (**F**, **L**, **R**) Differential distribution of *CCDC170, IRE1, XBP1* in the breast cancer groups with different TNM stages. The expression of *CCDC170*(**S**, **T**), IRE1(**U**, **V**) and *XBP1*(**W**, **X**) was not significantly associated with the overall survival and disease-free survival (based on the median). Datasets from TCGA were implemented for analysis. ER, estrogen receptor α; PR, progesterone receptor; Her2, human epidermal growth factor receptor 2; -, negative; +, positive; LN, lymph node status. The error bars presented as mean ± Standard Error of Mean (SEM) with analysis of Kruskal-Wallis test and Mann-Whitney test. $P < 0.05$ (two-tailed) was considered as statistically significant.

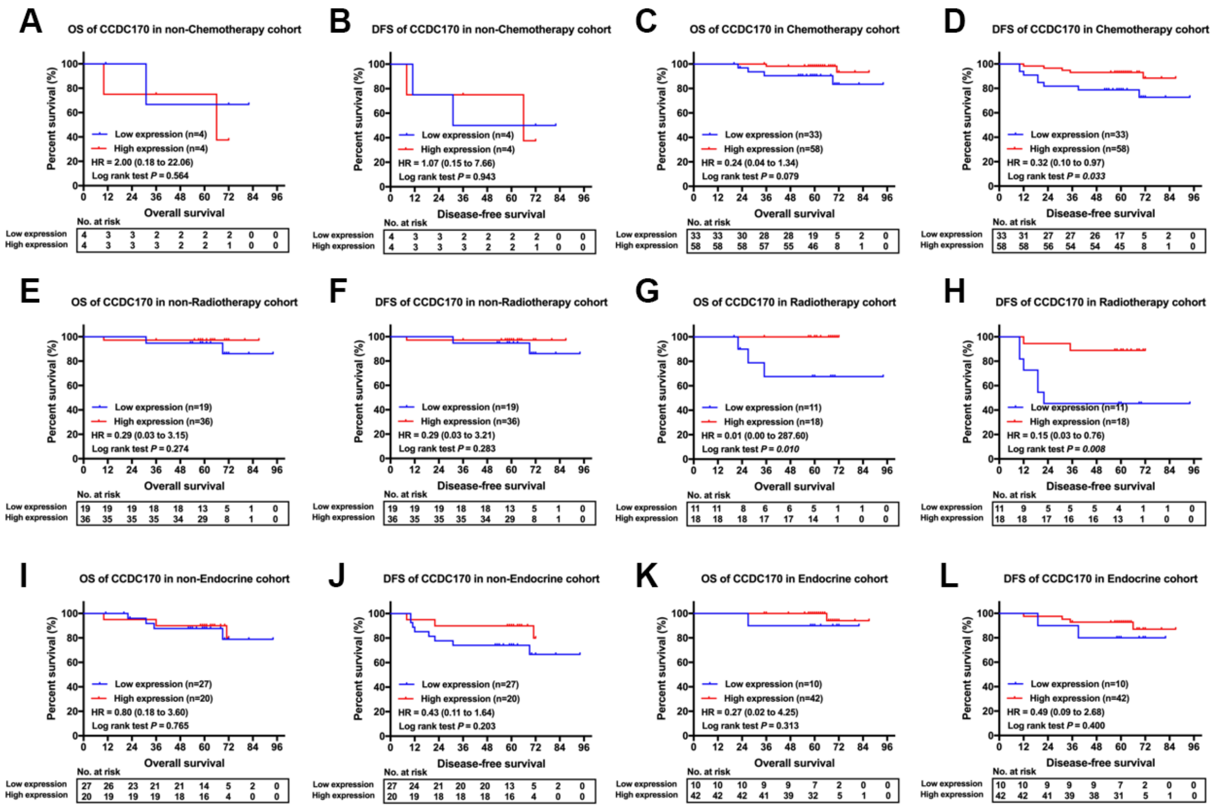

Supplementary Figure 5. The prognosis value of CCDC170 in breast cancer patients that received the same treatment. (**A**, **B**) The expression of CCDC170 showed no significant effect on the overall survival and disease-free survival in non-Chemotherapy cohort. (**C**, **D**) The CCDC170 high-expression group exhibited better disease-free survival in Chemotherapy cohort, but no significance for overall survival. (**E**, **F**) The expression of CCDC170 showed no significant effect on the overall survival and disease-free survival in non-Radiotherapy cohort. (**G**, **H**) Higher expression of CCDC170 predicted better overall survival and disease-free survival in Radiotherapy cohort. (**I**, **J**) The expression of CCDC170 showed no significant effect on the overall survival and disease-free survival in non-endocrine therapy cohort. (**K**, **L**) The expression of CCDC170 showed no significant effect on the overall survival and disease-free survival in endocrine therapy cohort. *P* < 0.05(two-tailed) was considered as statistically significant.
