# Supplementary material for: CCDC170 affects breast cancer apoptosis through IRE1 pathway
Source: Aging (Albany NY). 2020 Dec 3;13(1):1332–56. doi: 10.18632/aging.202315 (PMC7835043; doi:10.18632/aging.202315)
Supplement: Supplementary Tables [file aging-13-202315-s002.pdf]

SUPPLEMENTARY TABLES

Supplementary Table 1. The general situations of all patients complying with inclusive criteria.

Feature		case(n)	percent
Age(year)			
	≤ 30	2	2%
	31-40	12	12%
	41-50	33	33%
	51-60	30	30%
	61-70	16	16%
	≥ 71	7	7%
BMI			
(kg/m^2)	≤ 18.5	6	6%
	18.6-23.0	29	29%
	23.1-30.0	52	52%
	≥ 30.1	10	10%
	Non-available	3	3%
Menarche age			
	< 13	11	11%
	≥ 13	89	89%
Menopause Status			
	No	47	47%
	Yes	53	53%
Primitive age			
	< 28	61	61%
	≥ 28	29	29%
Number of live births			
	0	3	3%
	1	56	56%
	≥ 2	39	39%
History of breast cancer			
	No	82	82%
	Yes	18	18%
Tumor location			
	left	46	46%
	right	54	54%
Tumor size(d/cm)			
	≤ 2	38	38%
	2-5	56	56%
	> 5	6	6%
Lymph node metastasis			
	0	49	49%
	1-3	22	22%
	4-9	15	15%
	≥ 10	14	14%
TNM stage			
	I	34	34%
	II	37	37%
	III	29	29%
grade			
	I	5	5%
	II	61	61%
	III	18	18%
	Non-available	16	16%

ER			
	-	45	45%
	+	55	55%
PR			
	-	57	57%
	+	43	43%
Her-2			
	-	71	71%
	+	29	29%
Ki-67			
	≤ 14%	19	19%
	14 - 30%	26	26%
	≥ 30%	55	55%
Molecular subtype			
	Luminal A	16	16%
	Luminal B	48	48%
	HER2	15	15%
	TNBC	21	21%
Postoperative chemotherapy			
	No	8	8%
	Yes	91	91%
	Non-available	1	1%
Postoperative radiotherapy			
	No	55	55%
	Yes	29	29%
	Non-available	16	16%
Endocrine therapy			
	No	47	47%
	Yes	52	52%
	Non-available	1	1%

ER, estrogen receptor α; PR, progesterone receptor; Her-2, human epidermal growth factor receptor 2; TNBC, triple negative breast cancer; HER2, Her-2 overexpressed breast cancer; -, negative; +, positive.

Supplementary Table 2. The general situations of 732 patients from TCGA data.

Feature		case(n)	percent
Age(year)			
	≤ 30	12	1.6%
	31-40	69	9.4%
	41-50	161	22.0%
	51-60	190	26.0%
	61-70	181	24.7%
	≥ 71	119	16.3%
Menopause Status			
	No	166	24.4%
	Yes	496	73.0%
	Non-available	17	2.5%
History of breast cancer			
	No	693	94.7%
	Yes	38	5.2%
	Non-available	1	0.1%
Tumor location			
	left	398	54.4%
	right	334	45.6%
Tumor size(d/cm)			
	≤ 2	215	29.4%
	2-5	441	60.2%
	> 5	76	10.4%
Lymph node metastasis			
	0	344	47.0%
	1-3	264	36.1%
	4-9	87	11.9%
	≥ 10	30	4.1%
	Non-available	7	1.0%
TNM stage			
	I	134	18.3%
	II	443	60.5%
	III	155	21.2%
Margin status			
	No	630	86.1%
	Yes	42	5.7%
	Non-available	60	8.2%
ER			
	-	194	26.5%
	+	499	68.2%
	Non-available	39	5.3%
PR			
	-	264	36.1%
	+	431	58.9%
	Non-available	37	5.1%
Her-2			
	-	521	71.2%
	+	113	15.4%
	Non-available	98	13.4%
Pam50 subtype			
	Luminal A	164	22.4%
	Luminal B	289	39.5%
	HER2	109	14.9%

Basal like	163	22.3%
Normal-breast like	7	1.0%

ER, estrogen receptor α; PR, progesterone receptor; Her-2, human epidermal growth factor receptor 2; HER2, Her-2 overexpressed breast cancer; -, negative; +, positive.

Supplementary Table 3. The general situations of 3409 patients from GSE96058 datasets.

Feature		case(n)	percent
Age(year)			
	≤ 30	14	0.4%
	31-40	138	4.0%
	41-50	567	16.0%
	51-60	646	18.9%
	61-70	1100	32.3%
	≥ 71	944	27.7%
Lymph node metastasis			
	0	2099	61.6%
	1-3	897	26.3%
	≥ 3	312	9.2%
	Non-available	101	3.0%
Grade			
	1	505	14.8%
	2	1593	46.7%
	3	1246	36.6%
	Non-available	65	1.9%
ER			
	-	254	7.5%
	+	2935	86.1%
	Non-available	220	6.5%
PR			
	-	407	11.9%
	+	2644	77.6%
	Non-available	358	10.5%
Her-2			
	-	2843	83.4%
	+	438	12.8%
	Non-available	128	3.8%
Ki67			
	Low	651	19.1%
	High	940	27.6%
	Non-available	1818	53.3%
Pam50 subtype			
	Luminal A	1709	50.1%
	Luminal B	767	22.5%
	HER2	348	10.2%
	Basal like	360	10.6%
	Normal-breast like	225	6.6%

ER, estrogen receptor α; PR, progesterone receptor; Her-2, human epidermal growth factor receptor 2; HER2, Her-2 overexpressed breast cancer; -, negative; +, positive.
